# Identification of key genes related to metabolic cell death in hepatic ischemia-reperfusion injury from transcriptome data and mechanism research using single-cell data

**DOI:** 10.3389/fimmu.2025.1695979

**Published:** 2026-01-02

**Authors:** HongLi Yu, YingLi Cao, Jiebo Wang, Xianfeng Weng, Weituan Xu

**Affiliations:** 1 Department of Anesthesiology, Tianjin First Center Hospital, Tianjin, China; 2 Department of Anesthesiology, Peking University Third Hospital, Beijing, China; 3 Department of Anesthesiology, Fujian Medical University Union Hospital, Fuzhou, China

**Keywords:** drug prediction, hepatic ischemia-reperfusion injury, key genes, metabolic cell death, single-cell RNA sequencing

## Abstract

**Background:**

Ferroptosis and cuproptosis are closely associated with hepatic ischemia-reperfusion injury (HIRI). However, the significance of metabolic cell death-related genes (MRGs) in HIRI still awaits exploration. This study examined the molecular mechanisms through which key genes contribute to metabolic cell death in HIRI.

**Methods:**

GSE12720, GSE14951, and GSE171539 datasets and 478 MRGs were included. First, candidate genes were screened through differential expression analysis combined with MRGs. Then, key genes were identified by using machine learning algorithms combined with expression verification. Subsequently, the analyses included constructing and evaluating nomograms, conducting functional enrichment studies, characterizing immune infiltration, building regulatory networks, performing drug prediction, and executing molecular docking. Importantly, single-cell analysis was conducted to identify key cell populations Finally, expression levels of key genes in animal samples were determined by reverse transcription quantitative polymerase chain reaction (RT-qPCR).

**Results:**

The analysis identified ATF3, TNFAIP3, IL1B, and KDM6B as central genes. The nomogram indicated that these four key genes could well predict the occurrence of HIRI. Functional enrichment analysis revealed significant associations of ATF3, TNFAIP3, and KDM6B with olfactory transduction pathways. The key genes were positively linked to most differential immune cells, and ATF3 had the most significant positive relation to activated CD4 T cells. The binding energies of molecular docking between key genes and corresponding drugs were all less than -5 kcal/mol. Mononuclear phagocytes were identified as key cells, and the expressions of ATF3, IL1B, and KDM6B had dynamic and non-linear change characteristics during the differentiation of mononuclear phagocytes. RT-qPCR results demonstrated that ATF3, TNFAIP3, IL1B, and KDM6B were up-regulated in HIRI samples, consistent with the results in the GSE12720 and GSE14951 datasets.

**Conclusion:**

In HIRI pathogenesis research, ATF3, TNFAIP3, IL1B and KDM6B were validated as core regulators of metabolic cell death, offering critical targets for mechanistic investigation.

## 1 Introduction

Hepatic ischemia-reperfusion injury (HIRI) occurs when blood flow is temporarily interrupted and subsequently restored to liver tissue, triggering complex inflammatory cascades that paradoxically exacerbate cellular damage beyond the initial ischemic insult (1). Clinically manifested by elevated transaminases (ALT/AST), hepatocellular necrosis, and postoperative liver dysfunction, HIRI complicates more than 25% of liver resections and accounts for 29% of early graft failures in liver transplantation (2). The pathogenesis involves interdependent mechanisms: 1) Reactive Oxygen Species (ROS) bursts during reperfusion induce oxidative stress, which directly impairs mitochondrial function (3, 4); 2) Mitochondrial dysfunction further exacerbates ROS production and energy depletion (1); 3) These insults recruit neutrophils, whose mediated inflammation amplifies parenchymal damage (2).

Current therapeutic strategies remain limited to ischemic preconditioning and pharmacological agents like N-acetylcysteine (ROS scavenger) or rapamycin (autophagy inducer) (1), yet these exhibit suboptimal efficacy due to poor targeted delivery and failure to address spatial heterogeneity in zonal vulnerability (3). While engineered exosome therapies show promising reduction in transaminase levels (ALT decreased by 47.2%) in preclinical models (1), clinical translation remains constrained by unresolved molecular mechanisms underlying metabolic dysregulation—including ROS-mediated lipid peroxidation and mitochondrial energy failure (3, 4). This underscores the critical need to identify novel gene targets regulating cell-death pathways to develop precision interventions against HIRI’s multifaceted pathology.

Metabolically regulated cell death encompasses distinct pathways triggered by dysregulated accumulation of metal ions or metabolites, including ferroptosis (iron-dependent lipid peroxidation) and emerging forms like cuproptosis (copper-induced mitochondrial toxicity) (4). In HIRI, ferroptosis plays an experimentally validated role: CD4+ T cells drive hepatocellular ferroptosis through IFN-γ-dependent STAT1 activation, which promotes ACSL4-mediated lipid peroxidation and amplifies parenchymal damage (5). While cuproptosis has been implicated in neurological disorders via copper-induced FDX1 aggregation and mitochondrial respiration collapse (6), and dysregulated zinc homeostasis, known to disrupt lysosomal stability and trigger cell death (7), may also modulate hepatic injury responses during IRI, though this remains uncharacterized, these specific mechanisms remain uncharacterized in HIRI. Crucially, ferroptosis inhibitors (e.g., ferrostatin-1) reduce serum transaminases by more than 50% in HIRI models (5), underscoring the therapeutic potential of targeting metabolic death regulators. However, HIRI’s multifaceted pathology (e.g., concurrent oxidative stress and immune activation) suggests that targeting ferroptosis alone may be insufficient, highlighting the need to explore other metabolically regulated cell death pathways. Specifically, the spatiotemporal dynamics of these pathways—particularly regarding copper/zinc homeostasis in the hepatic microenvironment—warrant deeper investigation to identify novel intervention targets.

This study identified ATF3, TNFAIP3, IL1B, and KDM6B as core regulators of metabolic cell death in HIRI, which orchestrate inflammatory signaling, immune modulation, and epigenetic reprogramming. Key findings were validated using transcriptomic datasets (GSE12720, GSE14951), single-cell RNA sequencing (scRNA-seq) (GSE171539), and murine models, which also identified mononuclear phagocytes established as the central cellular hub. This study delineates a novel mechanistic axis in hepatic injury. A clinical nomogram incorporating these key genes exhibited robust diagnostic capability, complemented by molecular docking that identified promising therapeutic compounds targeting these regulators. These findings provide precise intervention targets and deepen the mechanistic comprehension of metabolic cell death.

## 2 Materials and methods

### 2.1 Data resources

Datasets related to HIRI were acquired via the Gene Expression Omnibus (GEO) database (http://www.ncbi.nlm.nih.gov/geo/). In this study, the GSE12720 microarray data (GPL570 platform) were assigned as the training set, containing 21 HIRI-affected liver specimens and 21 normal counterparts (termed HIRI and control cohorts for subsequent analysis). We employed GSE14951 (GPL570) as our validation set, comprising liver samples from 5 HIRI cases and 5 control individuals. In addition, GSE171539 (GPL20795) included scRNA-seq data of liver tissue samples from 5 HIRI patients and 1 control. By combining and removing duplicates of ferroptosis-related genes retrieved from the Ferroptosis Database (FerrDb) (http://www.zhounan.org/ferrdb/current/), and of cuproptosis-related, lysozincrosis-related, disulfidptosis-related, and alkaliptosis-related genes retrieved from the literature (8–10), 478 metabolism-related genes were obtained (**Supplementary Table 1**).

### 2.2 Recognition of candidate genes

The GSE12720 dataset was subjected to limma-based differential expression analysis (v3.54.0) (11), identifying transcripts with |log_2_ fold-change| > 0.5 and adjusted P-value < 0.05 in HIRI samples relative to controls. Additionally, We employed ggplot2 (v3.4.4) (12) to generate volcano plots and pheatmap (v1.0.12) (13) to construct heatmaps for visualizing the differential gene expression patterns. The VennDiagram R package (v1.7.1) (14) was employed to visualize and analyze the overlapping regions between differentially expressed genes and MRGs datasets, facilitating candidate gene.

### 2.3 Functional analysis of candidate genes

To elucidate candidate gene functions, we conducted Gene Ontology (GO) term and Kyoto Encyclopedia of Genes and Genomes (KEGG) pathway enrichment analyses with the clusterProfiler package (v4.7.1.003) (15), applying a significance threshold of P < 0.05. Subsequently, the protein-protein interaction (PPI) networks were generated from the STRING database (confidence score threshold > 0.4) and subsequently visualized using Cytoscape software (version 3.9.1) (16). Finally, chromosomal mapping of candidate genes was performed using the RCircos package (version 1.2.2) (17) to identify their genomic locations across human chromosomes.

### 2.4 Screening of key genes and analysis of their expression specificity

In accordance with candidate genes, the least absolute shrinkage and selection operator (LASSO) regression analysis was performed utilizing the glmnet (v 4.1-4) package (18). 10-fold cross-validation was applied to determine the optimal lambda value. Genes that were not penalized to 0 were regarded as LASSO genes. Meanwhile, Feature selection was performed through support vector machine-recursive feature elimination (SVM-RFE) analysis implemented in the caret package (version 6.0-93) (19). The accuracy of each combination was obtained, and the combination with the highest accuracy rate was selected as the optimal one to identify the SVM-RFE genes. The final feature genes were yielded by means of intersecting the LASSO genes and SVM-RFE genes utilizing the ggvenn (v 0.1.9) package (20). Subsequently, differential expression of the identified feature genes between GSE12720 and GSE14951 datasets was assessed using Wilcoxon rank-sum test, with statistical significance set at P < 0.05 for key gene identification. Additionally, Inter-gene correlations among the identified key genes were evaluated using Spearman’s rank correlation analysis implemented in the psych package (version 2.4.3) (21), with significant associations defined as |cor| > 0.3 at P < 0.05. Protein sequences corresponding to the key genes were acquired from the National Center for Biotechnology Information (NCBI) database (https://www.ncbi.nlm.nih.gov/), followed by subcellular localization prediction using the mRNALocater database (https://ngdc.cncb.ac.cn/databasecommons/database/id/1810). The Genotype-Tissue Expression (GTEx) resource (https://www.gtexportal.org/home/) was employed to evaluate the tissue distribution of key gene expression patterns in human biological systems.

### 2.5 Development and assessment of nomograms

To validate the predictive reliability of key genes for HIRI, we developed a prognostic nomogram based on GSE12720 dataset using the rms package (version 6.5-0) (22). Next, to evaluate the effectiveness of the nomogram, a calibration curve was produced via the rms package (v 6.5-0) (with P < 0.05); decision curve analysis (DCA) was carried out via the ggDCA package (v 1.2) (23); and a receiver operating characteristic (ROC) curve was plotted via the pROC package (v 1.18.5) (24) (area under the curve (AUC) > 0.7).

### 2.6 Gene set enrichment analysis

GSEA of the GSE12720 dataset was conducted to elucidate the functional pathways associated with key genes in HIRI. The c2.cp.kegg.v7.4.symbols.gmt gene set was obtained from the Molecular Signatures Database (MSigDB) (https://www.gsea-msigdb.org/gsea/msigdb/) as the reference background collection. Primary analysis involved assessing gene-gene correlations through Spearman’s rank-order method implemented in the psych package (version 2.4.3). GSEA was subsequently performed for individual key genes using clusterProfiler (version 4.7.1.003), applying significance thresholds of |Normalized Enrichment Score (NES)| > 1 and P < 0.05. The five most statistically significant pathways (ranked by ascending P-value) were visualized with the enrichplot package (v1.18.0) (25). In addition, Functional annotation of key genes was further expanded using the GeneMANIA platform (http://genemania.org), which predicts functionally associated genes and constructs gene-gene interaction (GGI) networks.

### 2.7 Immune infiltration analysis

Primary immune profiling of the GSE12720 dataset was performed using single-sample gene set enrichment analysis (ssGSEA) implemented in the GSVA package (version 1.50.0) (26), quantifying 28 immune cell subtypes (significance threshold P < 0.05). Resultant immune infiltration patterns were visualized via the pheatmap package (v1.0.12). Comparative analysis of immune cell infiltration between HIRI and control groups was performed using Wilcoxon rank-sum test (significance threshold P < 0.05), with results visualized through the ggpubr package (version 0.6.0) (27). Finally, correlations among the differential immune cells were explored utilizing the corrplot (v 0.92) package (13, 13) (|cor| > 0.30, P < 0.05), correlations between the differential immune cells and the key genes were explored utilizing the psych (v 2.2.9) package (|cor| > 0.30, P < 0.05), and heatmaps were drawn for visualization utilizing the ggcorrplot (v 0.1.4) package.

### 2.8 Construction of regulatory network

To investigate the molecular regulatory mechanisms of key genes, microRNAs (miRNAs) targeting these key genes were predicted using the multiMiR package (v 1.20.0) (28) based on the miRDB and miRanda databases. Intersection analysis was performed on the prediction outcomes from the two databases to derive miRNAs in the intersection. Subsequently, transcription factors (TFs) regulating the key genes were predicted in the NetworkAnalyst database (http://www.networkanalyst.ca), and a TF-key gene-miRNA network was formed.

### 2.9 Drug forecastin and molecular docking

Drugs that target the key genes were predicted using the Comparative Toxicogenomics Database (CTD) (https://ctdbase.org/). Drugs ranking in the top 5 for Interaction Counts associated with each key gene were selected to establish the drug-key gene network, which was visualized with Cytoscape software (v 3.9.1). Subsequently, using the CB-Dock database (https://cadd.labshare.cn/cb-dock/php/index.php), molecular docking analyses were performed on each key gene and its matching drug—with the highest Interaction Count and possessing a 3-dimensional (3D) structure in the PubChem database (https://pubchem.ncbi.nlm.nih.gov). Key genes were submitted to the Protein Data Bank (PDB) (https://www.rcsb.org/) for retrieving their protein 3D structures. Binding energies below -5 kcal/mol were deemed to possess strong binding capacity.

### 2.10 scRNA-seq analysis

At the outset, the GSE171539 dataset was handled using the Seurat package (v 5.0.1) (29). Quality filtering was applied to remove cells expressing fewer than 200 genes (min.features=200) and genes detected in less than 3 cells (min.cells=3). Next, high-quality cells and genes were further filtered based on more stringent criteria (600 < nFeature_RNA < 3000, 500 < nCount_RNA < 12000, percent.mt < 10%), and quality-control plots were generated using the VlnPlot function. The filtered single-cell data were normalized utilizing the NormalizeData function (scale factor = 10000). The top 2000 highly variable genes (HVGs) were selected utilizing the FindVariableFeatures function, and the top 5 HVGs were visualized and presented utilizing the VariableFeaturePlot function. Subsequently, principal component analysis (PCA) was conducted for the identified HVGs using runPCA function (P < 0.05).A scatter plot was drawn utilizing the DimPlot function to evaluate the cell distribution between HIRI and control samples. The JackStraw function was utilized to assess the contribution of the principal components (PCs) through significance testing, and 100 repeated calculations were conducted to enhance the stability of the results. The significant results were visualized utilizing the JackStrawPlot function. Next, the cumulative contribution of the PCs to the overall data variation was evaluated utilizing the Elbowplot function to determine the appropriate number of principal components for downstream analysis, and a scree plot was drawn for visualization. Following this, with the t-distributed stochastic neighbor embedding (t-SNE) clustering strategy, the FindNeighbors function was utilized to evaluate cell-to-cell similarities, whereas the FindClusters function was used to classify cells into separate clusters (resolution = 0.1). The cell clustering was visualized utilizing the DimPlot function. Afterward, each cell cluster was identified utilizing the marker genes from the literature (30) (**Supplementary Table 2**). Annotation results were visualized using the DimPlot function, while marker gene expression across different cell types was investigated using the DotPlot function.

### 2.11 Cell communication analysis and identification of key cells

Intercellular communication networks were reconstructed using the GSE171539 dataset (HIRI vs controls) through CellChat (version 1.6.1) (31), with receptor-ligand interactions systematically characterized via the integrated netVisual heatmap function. Furthermore, the proportions of annotated cell populations across HIRI and control samples were computed individually within the GSE171539 dataset, with visualization conducted using the ggplot2 package (v 3.4.4). Subsequently, key genes’ expression in all cell types was analyzed utilizing the FeaturePlot function in the Seurat (v 5.0.1) package. Violin plots showing the expression of key genes in each cell type were generated using the VlnPlot function, and differences in key gene expression between HIRI and control samples across each cell type were analyzed (P < 0.05) to pinpoint key cells.

### 2.12 Pseudotime analysis

To investigate key cell state transition processes and forecast the differentiation trajectory of key cells, key cells were retrieved from the GSE171539 dataset, with subsequent secondary dimensionality reduction and clustering performed, and these key cells were categorized into distinct cell subtypes (resolution = 0.1). Subsequently, to probe the relationship between the expression changes of key genes and the differentiation of key cells, cell pseudotime trajectory analysis was performed utilizing the Monocle (v 2.26.0) package (32), and visualization was carried out utilizing the plot cell trajectory function.

### 2.13 Animal model

Mice were randomly assigned into the HIRI group (n = 5) and the control group (n = 5).
Strain: Male C57BL/6 mice (8 weeks old, 22 ± 2 g).
Housing: Standard Specific Pathogen-Free (SPF) conditions (22 °C, 12-h light/dark cycle), ad libitum access to food/water.
Randomization: Mice were randomly assigned to:
HIRI group (n = 5).
Sham control group (n = 5).
Hepatic Ischemia-Reperfusion Injury Model.
Anesthesia: Induction with 2% isoflurane (O_2_ carrier gas) for surgical plane maintenance9.
Laparotomy: Midline incision (1.5 cm) under sterile conditions.
Vascular Occlusion:
Partial (70%) hepatic ischemia induced by clamping the left and median hepatic pedicles using non-traumatic microvascular clamps[citation:1, citation:6].
Ischemia duration: 60 min10.
Reperfusion:
Clamps removed to restore blood flow (confirmed visually by liver re-colorization).
Reperfusion duration: 6 hours 10.
Sham Procedure: Identical laparotomy and organ exposure without vascular clamping.
Tissue Harvest:
Left liver lobe collected and divided:
30 mg fixed in 4% paraformaldehyde (histology).
Remainder flash-frozen in liquid N_2_ (-80 °C storage for RNA).

### 2.14 RT-qPCR

To further examine the expression of key genes in animal samples, RT-qPCR was conducted using liver tissue samples obtained from 5 HIRI mice and 5 control mice. The study had the approval of the ethics committee of Tianjin First Central Hospital (approval number:2020N198KY). Total RNA from the 10 samples was isolated using TRIzol reagent (Ambion, USA) following the manufacturer’s instructions. Subsequently, RNA concentration was determined using NanoPhotometer N50. cDNA was generated via reverse transcription using the SureScript-First-strand-cDNA-synthesis-kit, and reverse transcription was carried out using the S1000TM Thermal Cycler (Bio-Rad, USA). Primer sequences used in RT-qPCR are listed in **Supplementary Table 3**. RT-qPCR was conducted using a Bio-Rad CFX Connect system (USA) with the following thermal cycling parameters: initial denaturation at 95°C for 1 min, followed by 40 cycles of 95°C for 20 s, 55°C for 20 s, and 72°C for 30 s. Relative mRNA expression was computed through the 2-ΔΔCT calculation method. GAPDH was utilized as the internal reference gene. RT-qPCR data were processed in Microsoft Excel prior to statistical analysis and graphical representation using GraphPad Prism 5 software, with statistical significance set at P < 0.05.

### 2.15 Statistical analysis

Computational analyses were performed using R statistical software (version 4.2.2), with a significance threshold established at P < 0.05. For RT-qPCR data evaluation, Student’s t-test was applied for group comparisons.

## 3 Results

### 3.1 Candidate genes were identified and their functions explored

**Figure 1:**
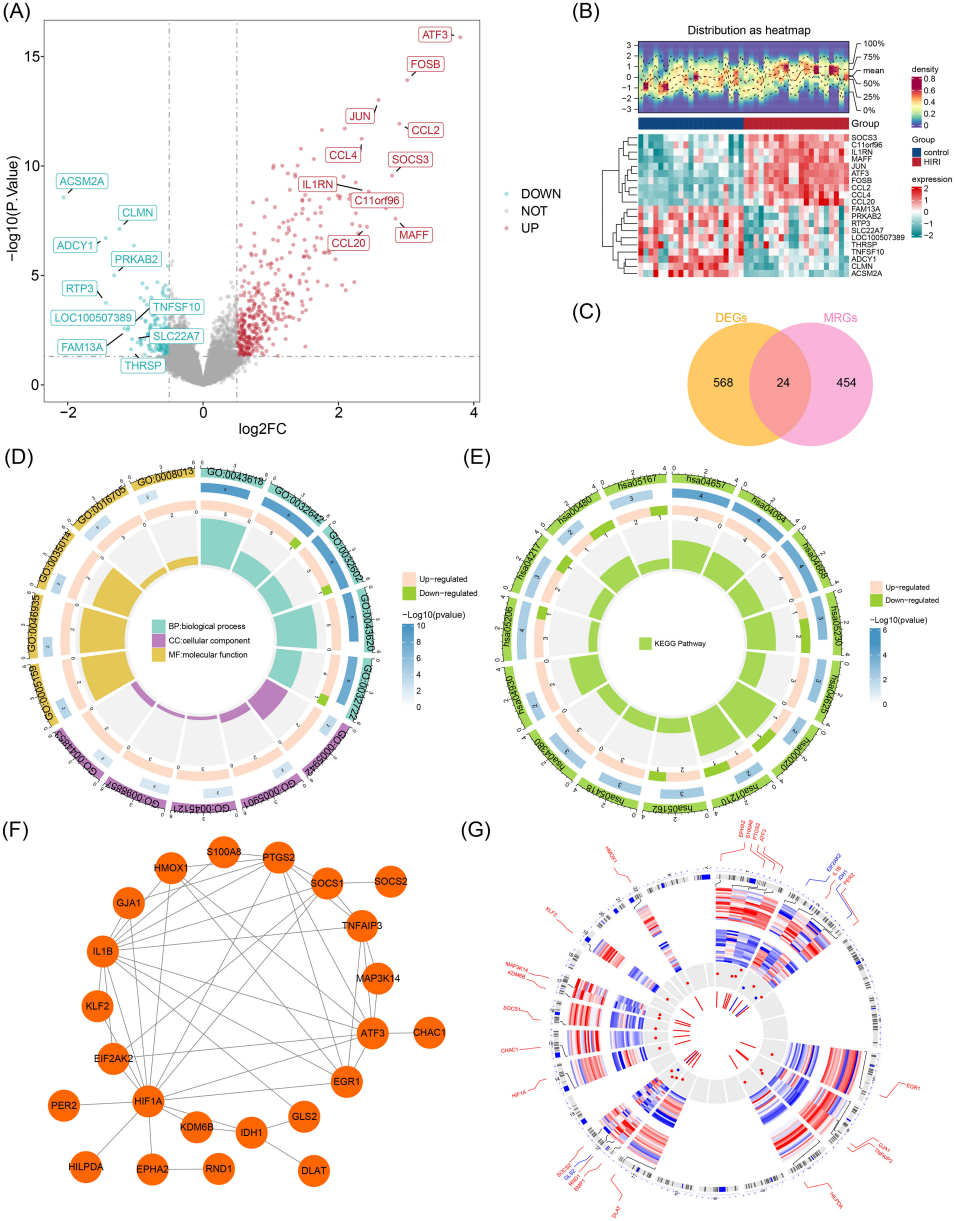
Identification and functional annotation of metabolism-related candidate genes in hepatic ischemia-reperfusion injury. **(A)** Volcano plot of DEGs in GSE12720 (|log_2_FC| > 0.5, P < 0.05). Red dots: 405 up-regulated genes; blue dots: 187 down-regulated genes. Top 10 DEGs labeled by gene symbol. **(B)** Heatmap of top 20 DEGs (rows) clustered by Euclidean distance (columns: samples). Color scale: red (high), blue (low). **(C)** Venn diagram intersection of 592 DEGs and 478 metabolism-related genes (MRGs), identifying 24 candidate genes. **(D)** GO enrichment for “regulation of chemokine production” (GO:0032642). Bar height: -log_10_(P-value). **(E)** KEGG pathway enrichment of NF-κB signaling pathway (hsa04064). Circle size: gene count; color: significance level. **(F)** Protein-protein interaction network (STRINGdb confidence score > 0.4), highlighting hub genes (HIF1A, IL1B, ATF3). One unconnected node excluded. **(G)** Chromosomal distribution showing EPHA2 (chr1:16,124,860-16,228,218), S100A8 (chr1:153,357,199-153,358,596), PTGS2 (chr1:186,671,791-186,683,549), and ATF3 (chr1:212,565,527-212,577,355) co-localization on chromosome 1 (GRCh38).Data source: GEO GSE12720. DEG screening: |log_2_FC| > 0.5, Benjamini-Hochberg adjusted *P* < 0.05. PPI network: STRING v11.0 (minimum interaction score 0.4). Enrichment significance: Fisher’s exact test *P* < 0.05. Gene positions: NCBI Genome Database.

Analysis of the GSE12720 dataset identified 592 DEGs meeting the threshold criteria of |log_2_ fold-change| > 0.5 and P < 0.05, comprising 405 up-regulated and 187 down-regulated transcripts. The volcano plot (**Figure 1A**) and heatmap (**Figure 1B**) highlighted the expression patterns of the top 10 most significantly up- and down-regulated DEGs, ranked by absolute log_2_ fold-change. Intersection analysis of the 592 DEGs and 478 MRGs identified 24 overlapping candidate genes (**Figure 1C**). Enrichment analysis pointed out that these 24 candidate genes were enriched in 906 GO terms (P < 0.05) (**Supplementary Table 4**), such as regulation of chemokine production (**Figure 1D**). In the KEGG, they were enriched in 30 pathways (P < 0.05) (**Supplementary Table 5**), such as the NF-kappaB signaling pathway (**Figure 1E**). This demonstrates that the candidate genes are intimately linked to the regulation of immunity and inflammatory responses. After constructing the PPI network, 1 discrete target was removed (confidence score > 0.4) (**Figure 1F**). Genes such as HIF1A, IL1B, and ATF3 were highly correlated with other genes. In addition, among these candidate genes, EPHA2, S100A8, PTGS2, and ATF3 were all located on chromosome 1 (**Figure 1G**), suggesting that they may have similar biological functions.

### 3.2 ATF3, TNFAIP3, IL1B, and KDM6B were deemed as key genes

**Figure 2:**
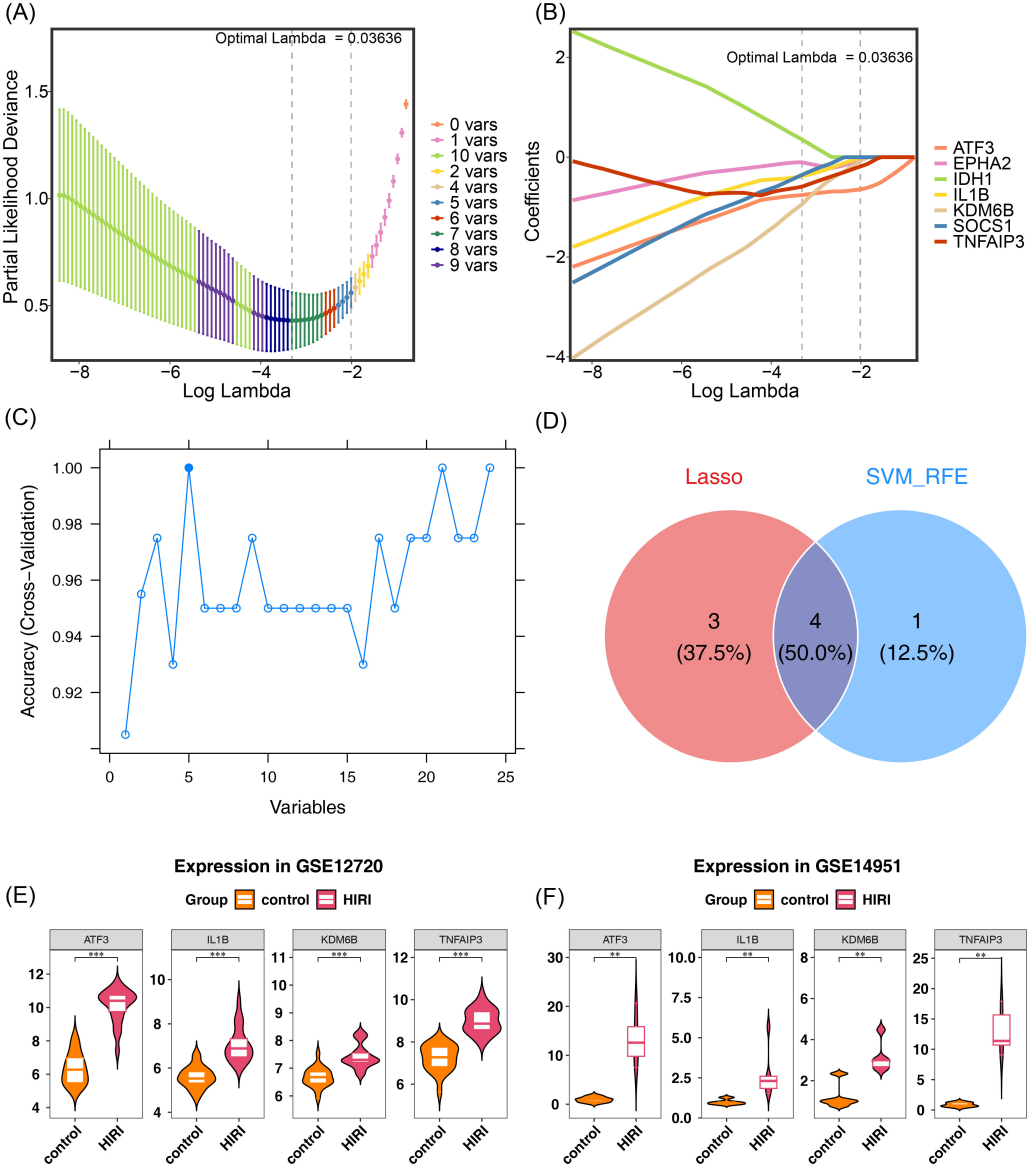
Machine learning-based identification and validation of HIRI key genes. **(A)** LASSO coefficient profiles of candidate genes. Dashed vertical line: optimal λ (λmin = 0.03636) yielding 7 genes. **(B)** LASSO cross-validation (10-fold) showing mean squared error (MSE) vs. log(λ). Red dot: λmin position. **(C)** SVM-RFE feature ranking (5-fold cross-validation). Dashed line: optimal feature subset (TNFAIP3, ATF3, RND1, IL1B, KDM6B) at peak accuracy. **(D)** Venn diagram intersecting LASSO (7 genes) and SVM-RFE (5 genes) outputs, identifying 4 consensus genes (ATF3, TNFAIP3, IL1B, KDM6B). **(E, F)** Validation of expression patterns for consensus genes in **(E)** GSE12720 and **(F)** GSE14951 datasets. Boxplots show significant upregulation in HIRI vs. controls (***P < 0.001, **P < 0.01, *P* < 0.05; t-test). *LASSO: R glmnet package (α=1). SVM-RFE: caret R package (linear kernel, cost=1). Statistical thresholds: P<0.05. GEO datasets: GSE12720 (mouse), GSE14951 (human). Gene symbols: HUGO nomenclature*.

**Figure 3:**
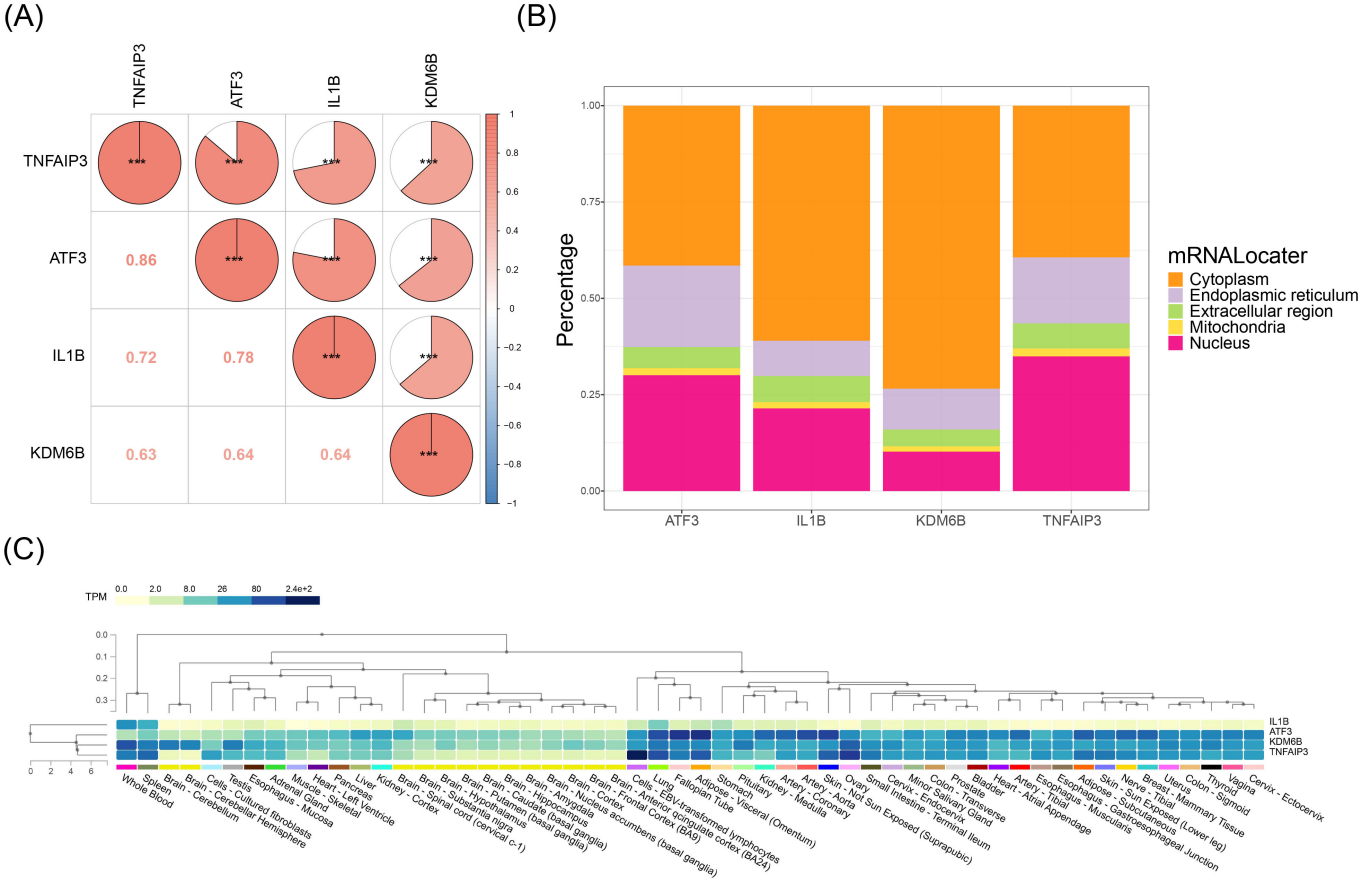
Functional characterization of key gene interactions and expression patterns. **(A)** Spearman correlation matrix of ATF3, TNFAIP3, IL1B, and KDM6B expression in HIRI samples (GSE12720 dataset). Color intensity and circle size reflect correlation strength (cor > 0.7: dark red; cor < -0.7: dark blue). ATF3-TNFAIP3 shows the strongest positive correlation (ρ = 0.86, *P* = 2.1e-05). **(B)** Subcellular localization predicted by the COMPARTMENTS database. Bar height indicates relative frequency across cellular compartments (cytoplasm: 49.3%-62.1%; nucleus: 27.6%-38.4%). **(C)** Tissue-specific expression (RNA-Seq data from GTEx). Heatmap colors: red (high), white (medium), blue (low). Asterisks mark tissues with TPM > 75th percentile: whole blood (IL1B, KDM6B), spleen (all genes), lung (TNFAIP3, IL1B), kidney medulla (ATF3, KDM6B). *Correlation significance: Benjamini-Hochberg adjusted P < 0.05. Subcellular data: experimental evidence confidence > 0.7 (COMPARTMENTS v2023). Tissue expression: GTEx v10 normalized TPM values*.

In the LASSO analysis, 7 LASSO genes were screened out based on a lambda min of 0.03636, including ATF3, TNFAIP3, IL1B, SOCS1, KDM6B, EPHA2, and IDH1 (**Figures 2A, B**). Meanwhile, 5 SVM-RFE genes were obtained from the SVM-RFE analysis based on the highest accuracy point, including TNFAIP3, ATF3, RND1, IL1B, and KDM6B (**Figure 2C**). Then, 4 feature genes were yielded by means of intersecting the 7 LASSO genes and the 5 SVM-RFE genes, namely ATF3, TNFAIP3, IL1B, and KDM6B (**Figure 2D**). Differential expression analysis revealed that ATF3, TNFAIP3, IL1B, and KDM6B exhibited statistically significant variations (P < 0.05) across both GSE12720 and GSE14951 datasets, and their expression was up-regulated in HIRI samples (**Figures 2E, F**). Therefore, ATF3, TNFAIP3, IL1B, and KDM6B were regarded as key genes. In addition, Spearman’s correlation analysis demonstrated that there were significant positive correlations among ATF3, TNFAIP3, IL1B, and KDM6B (**Figure 3A**). Among them, ATF3 and TNFAIP3 showed the most significant positive correlation (cor = 0.86, P < 0.05). Subcellular localization revealed that ATF3, TNFAIP3, IL1B, and KDM6B were mainly expressed in the cytoplasm, and their expression in the nucleus was also relatively high (**Figure 3B**). Expression specificity analysis indicated that ATF3, TNFAIP3, IL1B, and KDM6B were highly expressed in whole blood, spleen, and lung. Moreover, ATF3, TNFAIP3, and KDM6B were also highly expressed in various tissues, such as the fallopian tube, stomach, pituitary, and kidney medulla (**Figure 3C**). These results provide fundamental insights for elucidating the functional significance of these genes in diverse biological pathways.

### 3.3 Development and validation of key gene-based nomogram

**Figure 4:**
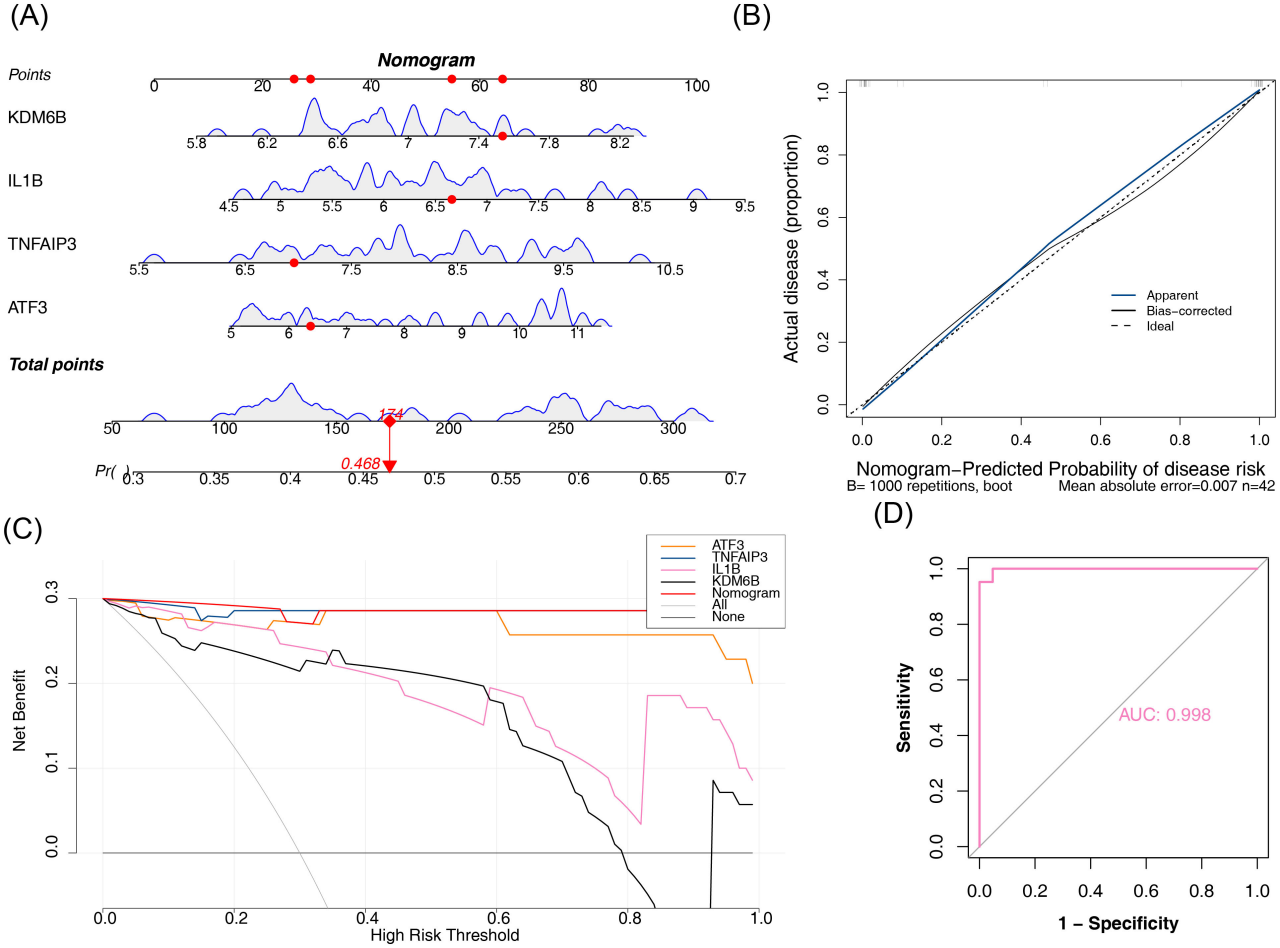
Development and validation of a key gene-based nomogram for HIRI prediction. **(A)** Nomogram integrating expression levels of ATF3, TNFAIP3, IL1B, and KDM6B. Point assignments: upper axis; total points → HIRI probability: lower axis. **(B)** Calibration curve (1000× bootstrap) comparing predicted vs. observed probabilities. Solid line: model performance (mean absolute error = 0.007); dashed line: ideal fit. **(C)** Decision curve analysis (DCA) showing net benefit across threshold probabilities (0.1–0.9). Red: key-gene model; gray: treat-all; black: treat-none. **(D)** ROC curve with AUC = 0.998 (95% CI: 0.992–1.000) in validation cohort (GSE14951). *Model development: the rms R package. Calibration: Harrell’s method. DCA: standardized net benefit calculation. AUC: DeLong’s test. Validation cohort: n=24 HIRI vs. 18 controls*.

Key gene-based nomogram construction yielded strong predictive performance for HIRI onset (**Figure 4A**), supported by high-fidelity calibration (Mean Absolute Error (MAE) = 0.007) between model predictions and actual observations (**Figure 4B**). Decision curve analysis demonstrated superior clinical net benefit of the integrated model compared to individual predictors (**Figure 4C**), while receiver operating characteristic analysis confirmed exceptional discriminative capacity (AUC = 0.998, **Figure 4D**). These results indicated that the nomogram of key genes had good predictive performance for the onset of HIRI.

### 3.4 Functional enrichment helped explore the potential mechanism for HIRI

**Figure 5:**
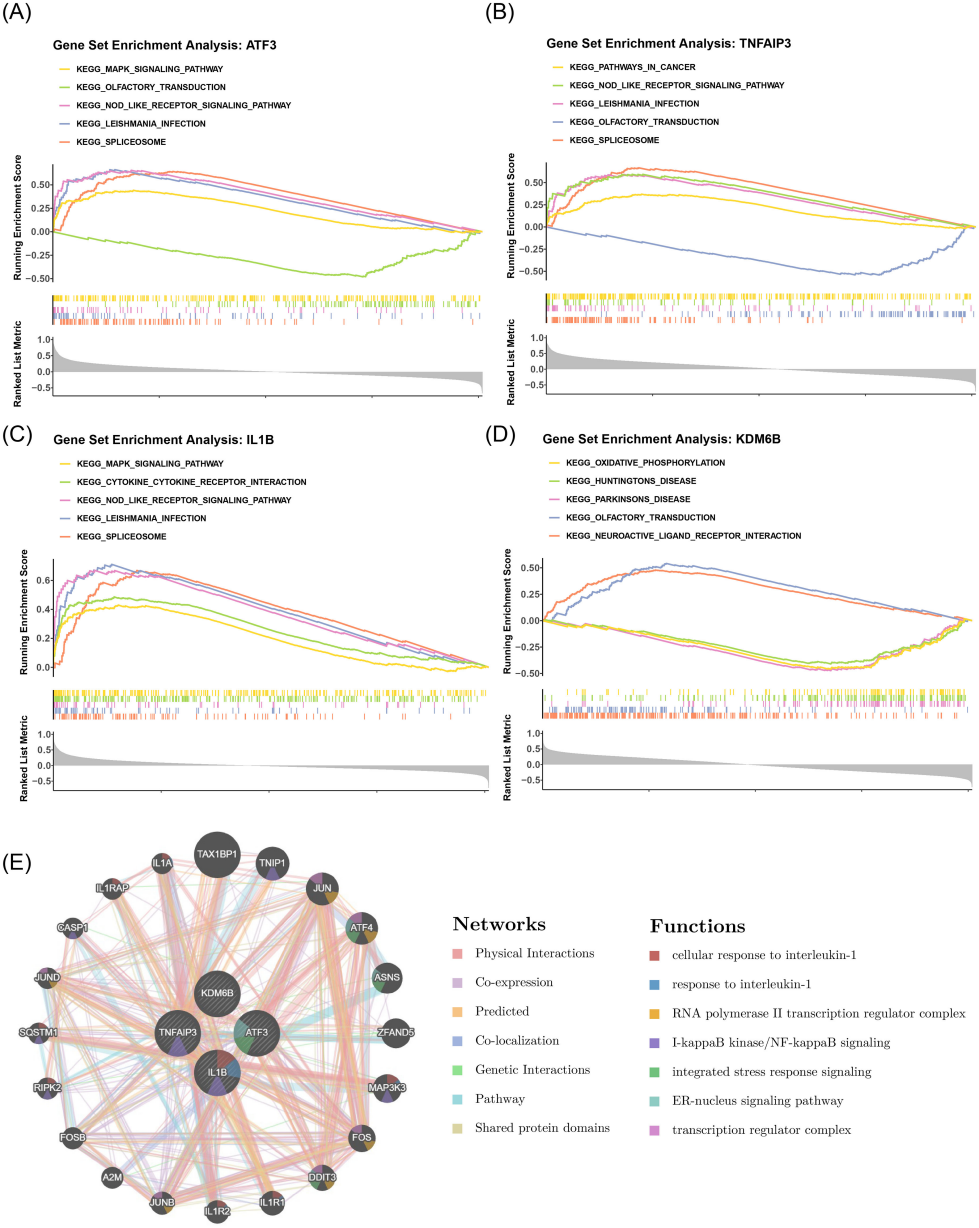
Pathway enrichment analysis and gene-gene interaction networks of HIRI key genes. **(A-D)** GSEA enrichment plots for **(A)** ATF3, **(B)** TNFAIP3, **(C)** IL1B, and **(D)** KDM6B. Top pathways: spliceosome (NES = 2.41, FDR<0.001), Leishmaniasis (NES = 2.35, FDR = 0.004), NOD-like receptor signaling (NES = 2.18, FDR = 0.008), olfactory transduction (NES = 2.62, FDR<0.001). Dashed lines: |NES|>1. **(E)** Gene-gene interaction network displaying top 20 functional partners (e.g., TAX1BP1). Gold edges: interactions with key genes; circle size: betweenness centrality. Enriched GO terms: transcription regulator complex (GO:0005667, FDR = 6.7e^-5^), cellular response to interleukin-1 (GO:0071347, FDR = 1.2e^-4^). *GSEA parameters: |NES|>1, FDR<0.05 (MSigDB C2 KEGG_2021). GGI network: STRINGdb (confidence>0.9), Cytoscape v3.9.1 (force-directed layout)*.

Gene set enrichment analysis identified significant pathway associations for ATF3 (59 pathways), TNFAIP3 (71 pathways), IL1B (58 pathways), and KDM6B (67 pathways) at stringent thresholds (|NES| > 1, P < 0.05; **Supplementary Table 6**). Notably, Pathway enrichment analysis demonstrated conserved functional patterns among key genes: ATF3, TNFAIP3 and IL1B co-enriched in spliceosome, Leishmania infection and NOD-like receptor signaling pathways, while ATF3, TNFAIP3 and KDM6B shared olfactory transduction involvement (**Figures 5A-D**). This indicated that these key genes might play roles in HIRI through inflammation and immune regulation mechanisms. In addition, The gene-gene interaction network identified 20 functionally relevant partners (including TAX1BP1) for the key genes, with enrichment in transcription regulator complexes and interleukin-1 response pathways (**Figure 5E**).

### 3.5 Key genes significantly linked with immune-infiltrating cells

**Figure 6:**
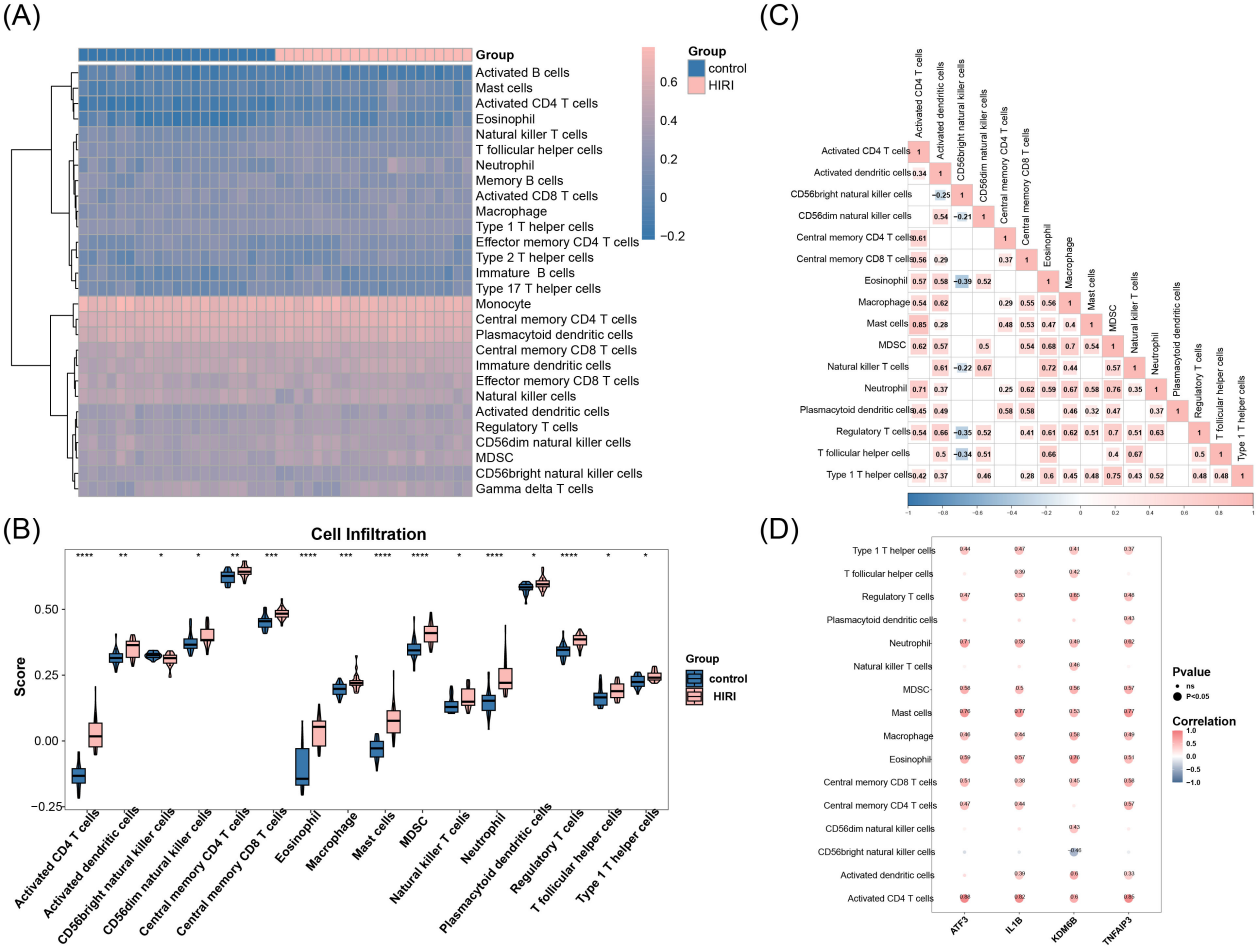
Immune cell infiltration landscape and key gene correlations in HIRI. **(A)** Heatmap of 28 immune cell types (rows) across HIRI vs. control samples (columns) quantified via CIBERSORTx. Color scale: red (high abundance), blue (low). **(B)** Differential infiltration of 16 cell types (*P* < 0.05, Benjamini-Hochberg corrected). Boxplots show CD56^bright^ NK cells (*P* = 3.2e^-4^) as representative. **(C)** Correlation network of altered immune cells. Gold edges: positive correlation (e.g., mast cells/activated CD4^+^ T cells: ρ = 0.85, *P* = 8.7e^-6^); blue edges: negative (eosinophils/CD56^bright^ NK cells: ρ = -0.39, P = 0.02). **(D)** Key gene-immunocyte correlations: Red: positive (max: ATF3/activated CD4^+^ T cells, ρ = 0.88, P = 2.1e^-6^); blue: negative (max: KDM6B/CD56^bright^ NK cells, ρ = -0.46, *P* = 0.005). *Deconvolution algorithm: CIBERSORTx (LM22 signature). Significance thresholds: FDR < 0.05, permutation tests (1000×). Correlation method: Spearman’s rank with Bonferroni adjustment. Sample cohort: GSE12720 (n=37 HIRI, n=20 controls)*.

**Figure 6A** showed the infiltration levels of 28 types of immune cells in HIRI and control samples. Among these, infiltration levels of 16 immune cell types differed significantly (P < 0.05; **Figure 6B**), including CD56bright natural killer (NK) cells. Furthermore, correlations between differential immune cells revealed that eosinophils exhibited the most pronounced negative correlation with CD56bright NK cells (cor = -0.39, P < 0.05), while mast cells displayed the most marked positive correlation with activated CD4 T cells (cor = 0.85, P < 0.05; **Figure 6C**). Key genes exhibited positive correlations with the majority of differential immune cells. Among them, ATF3 had the most significant positive correlation with activated CD4 T cells (cor = 0.88, P < 0.05), and KDM6B had the most significant negative correlation with CD56bright NK cells (cor = -0.46, P < 0.05) (**Figure 6D**).

### 3.6 Regulatory networks of key genes were established

**Figure 7:**
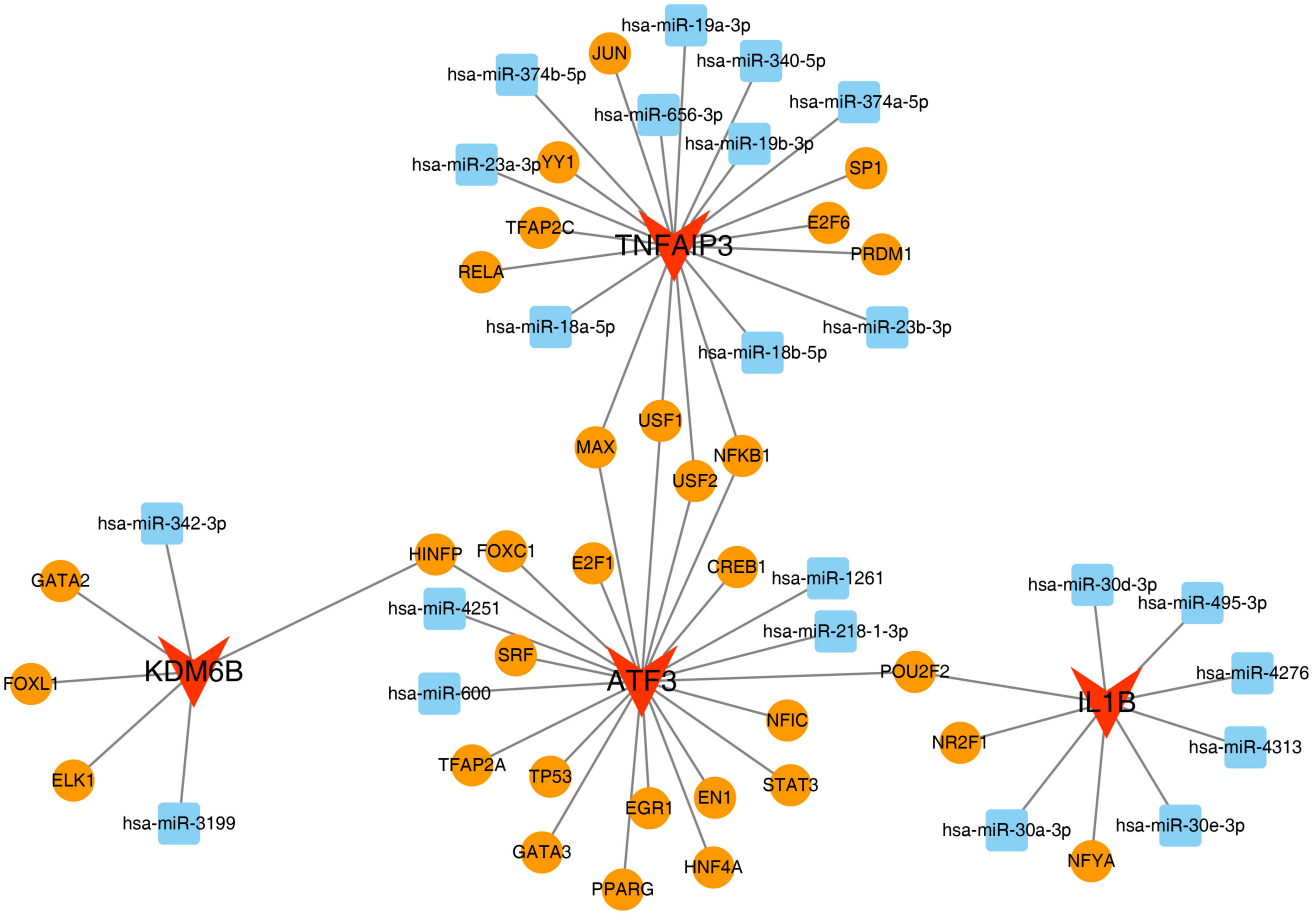
TF-key gene-miRNA regulatory network in hepatic ischemia-reperfusion injury. **(A)** miRNA screening: 22 miRNAs identified by intersecting predictions from miRDB (v6.0, score >80) and TargetScan (v8.0, context++ score <−0.3). **(B)** TF prediction: 31 TFs targeting key genes via NetworkAnalyst (v3.0; ENCODE ChIP-seq data): ATF3 (19 TFs), TNFAIP3 (11 TFs), IL1B (3 TFs), KDM6B (4 TFs). **(C)** Co-regulatory TFs: NFKB1, MAX, USF1/USF2 target both ATF3 and TNFAIP3; HINFP targets ATF3 and KDM6B; POU2F2 targets ATF3 and IL1B. **(D)** Integrated network: Gold diamonds (TFs), blue squares (key genes), red circles (miRNAs). Edge thickness scales with regulatory evidence score. Node size reflects connectivity (ATF3 degree=19 vs. IL1B degree=3). *TF prediction confidence: ENCODE uniform peak calls. miRNA-TF interactions prioritized by ≥2 database support. Network visualization: Cytoscape v3.9.1 (force-directed layout)*.

From the miRDB database, 88 miRNAs were identified as targeting the key genes, including 20 that target ATF3, 36 that target TNFAIP3, 12 that target IL1B, and 20 that target KDM6B. In the miRanda database, 98 miRNAs were retrieved to target the key genes, with 27 miRNAs targeting ATF3, 24 targeting TNFAIP3, 21 targeting IL1B, and 26 targeting KDM6B (**Supplementary Table 7**). Following the intersection of miRNAs predicted from the two databases, 22 overlapping miRNAs were identified. Subsequently, in the NetworkAnalyst database, 31 TFs were identified as targeting the key genes. Among them, 19 TFs were predicted to target ATF3, 11 target TNFAIP3, 3 target IL1B, and 4 target KDM6B. Specifically, NFKB1, MAX, USF1, and USF2 were found to be able to target both ATF3 and TNFAIP3 simultaneously, HINFP could target both ATF3 and KDM6B, and POU2F2 could target both ATF3 and IL1B. Based on these findings, a TF-key gene-miRNA network was constructed (**Figure 7**). The above results indicated that, compared with other key genes, ATF3 might play a more crucial role in HIRI.

### 3.7 Drug prediction and molecular docking targeting key genes were conducted

**Figure 8:**
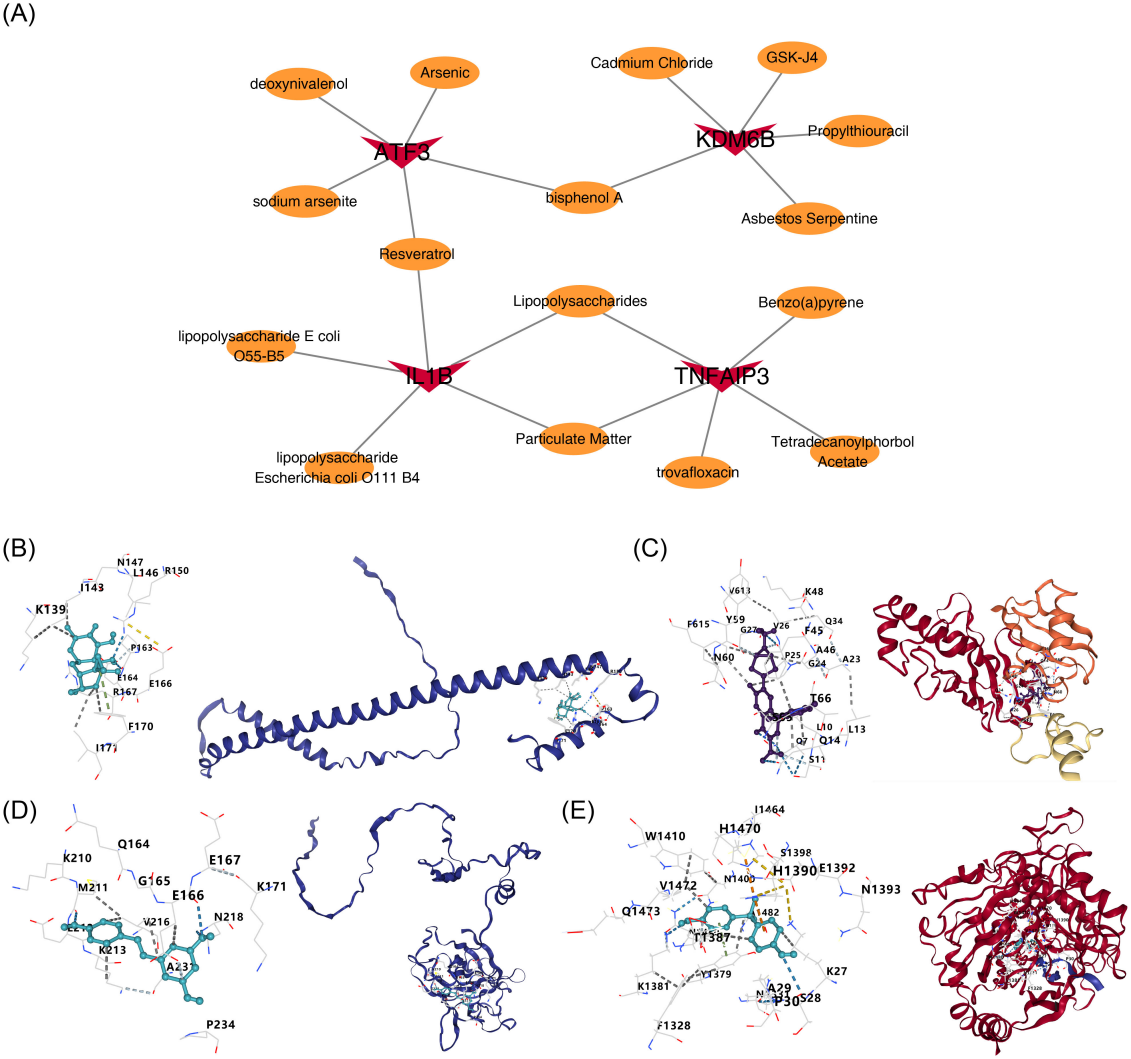
Drug targeting prediction and molecular docking validation of key HIRI genes. **(A)** Drug-key gene interaction network based on Comparative Toxicogenomics Database (CTD). Nodes: Gold circles (key genes), blue squares (drugs; top 5 interaction count per gene). Multitarget drugs: Bisphenol A (CID: 6623) → ATF3/KDM6B; Resveratrol (CID: 445154) → ATF3/IL1B; Lipopolysaccharides/Particulate matter → TNFAIP3/KDM6B. **(B-E)** Molecular docking poses for optimal ligand-target pairs: **(B)** ATF3-Deoxynivalenol (CID: 40024): ΔG = -8.1 kcal/mol; **(C)** TNFAIP3-Trovafloxacin (CID: 5386): ΔG = -10.7 kcal/mol; **(D)** IL1B-Resveratrol: ΔG = -7.9 kcal/mol; **(E)** KDM6B-Bisphenol A: ΔG = -6.4 kcal/mol. Hydrogen bonds are shown as green dashes. *Drug screening: CTD (February 2024 update). Ligand preparation: PubChem 3D structures. Docking: AutoDock Vina 1.2.0 (grid center: binding pocket centroid). Binding affinity threshold: ΔG < -5 kcal/mol*.

In the CTD database, 509, 283, 2212, and 122 drugs were predicted to target ATF3, TNFAIP3, IL1B, and KDM6B, respectively (**Supplementary Table 8**). Drugs with the top 5 Interaction Counts corresponding to each key gene were utilized to construct a drug-key gene network (**Figure 8A**). Among them, bisphenol A was predicted to target both ATF3 and KDM6B, resveratrol was predicted to target both ATF3 and IL1B, and lipopolysaccharides (LPS) as well as particulate matter were predicted to target both TNFAIP3 and KDM6B. The drugs with the highest Interaction Counts corresponding to ATF3, TNFAIP3, IL1B, and KDM6B, which also had 3D structures in the PubChem database, were deoxynivalenol, trovafloxacin, resveratrol, and bisphenol A, respectively. Molecular docking simulations demonstrated strong binding affinities between the key genes and their predicted drug compounds, with all binding energies below -5 kcal/mol. TNFAIP3 had the best binding ability with trovafloxacin, and its binding energy was -10.7 kcal/mol (**Figures 8B-E**, **Table 1**). Collectively, these findings underscore the therapeutic potential of ATF3, TNFAIP3, IL1B, and KDM6B as molecular targets for intervention.

**Table 1:** The binding energies of drug-gene.

Gene	Pro ID	Molecule	Vina Score(kcal/mol)
ATF3	P18847	deoxynivalenol	-6.1
TNFAIP3	P21580	trovafloxacin	-10.7
IL1B	P01584	Resveratrol	-6.5
KDM6B	O15054	bisphenol A	-7.2

### 3.8 Cell annotation generated 5 cell types

**Figure 9:**
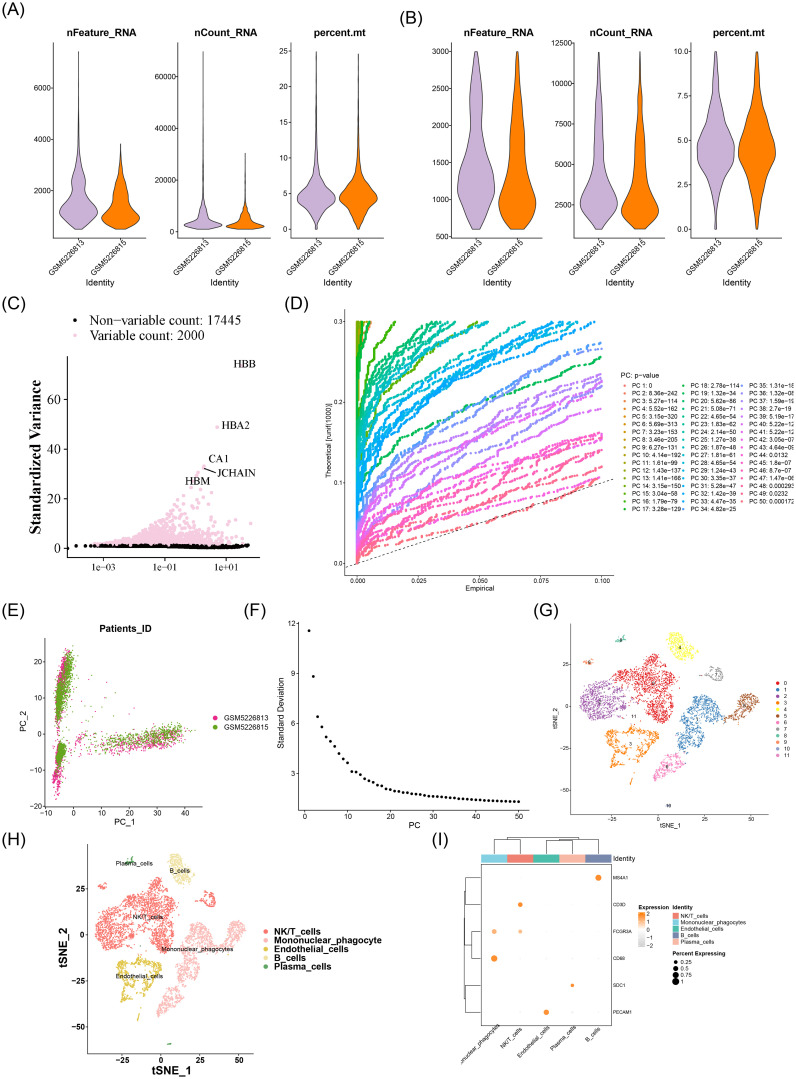
Single-cell transcriptomic profiling of hepatic ischemia-reperfusion injury. **(A, B)** Quality control metrics: **(A)** Pre-QC: 9,193 cells, 19,445 genes; **(B)** Post-QC (mitochondrial genes <10%, gene counts 200–5,000): 7,736 cells retained. **(C)** Highly variable gene (HVG) selection: Top 2,000 HVGs (variance-stabilizing transformation). **(D)** Principal component analysis: PCA plot shows significant separation between HIRI (red) and control (blue) groups (*P* < 0.05, PERMANOVA). **(E, F)** Dimensionality reduction: **(E)** Elbow plot of PC cumulative variance; **(F)** t-SNE clustering using top 20 PCs (perplexity=30). **(G)** Unsupervised clustering: 12 distinct cell clusters (resolution=0.8). **(H)** Cell-type annotation: Identified as mononuclear phagocytes (clusters 0/4/8; LYZ+), NK/T cells (clusters 1/5; NKG7+), B cells (clusters 2/6; CD79A+), endothelial cells (clusters 3/9; VWF+), and plasma cells (clusters 7/10; MZB1+). **(I)** Marker gene expression: Dot plot showing cell-type-specific signatures (CD68 for phagocytes, CD3D for T cells). *Data source: GSE171539. Analysis pipeline: Seurat v4.3.0. HVG selection: variance >0.5. Statistical threshold: PC significance (JackStraw P<0.05). Annotation references: PanglaoDB & CellMarker*.

Within the GSE171539 dataset, prior to quality control, the counts of cells and genes were 9,193 and 19,445, respectively. After quality control, 7,736 cells and 19,445 genes were retained (**Figures 9A, B**). Following quality control, 7,736 cells and 19,445 genes were kept (**Figure 9C**). Using these HVGs as a basis, the top 50 PCs were computed, and a difference in distribution was observed between the HIRI and control samples (P < 0.05) (**Figure 9D**). By evaluating the cumulative contribution of these PCs to the overall data, 20 appropriate PCs were selected for t-SNE clustering (**Figures 9E, F**). All high-quality cells were grouped into 12 distinct cell clusters (**Figure 9G**). The cell clusters were annotated, and 5 cell types were identified, including mononuclear phagocytes, NK/T cells, B cells, endothelial cells, and plasma cells (**Figure 9H**). Marker genes exhibited specificity to some extent across distinct cell clusters (**Figure 9I**).

### 3.9 Mononuclear phagocytes were identified as key cells

**Figure 10:**
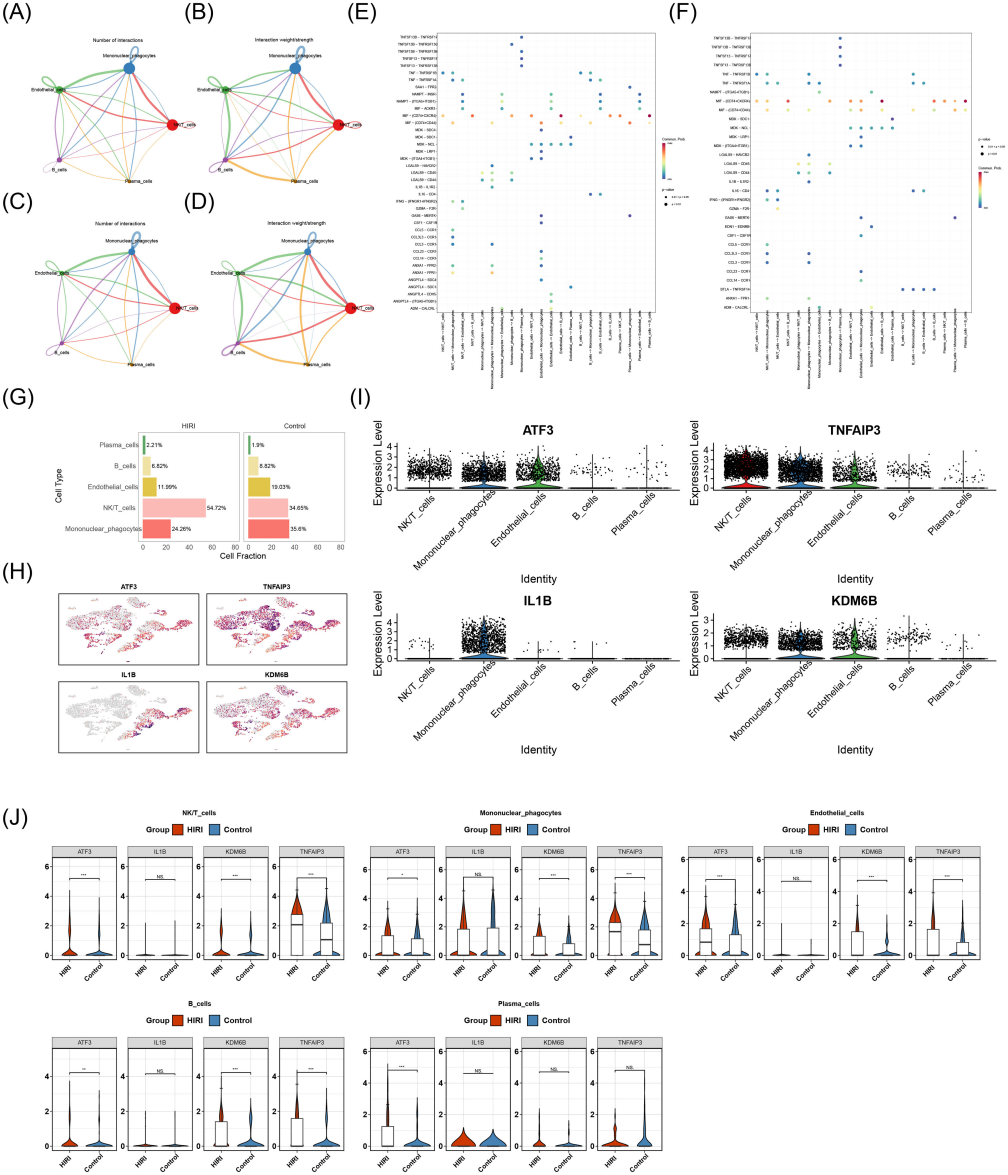
Cellular communication dynamics and key gene expression signatures in hepatic ischemia-reperfusion injury. **(A-D)** Cell-cell interaction networks: **(A, B)** Control and **(C, D)** HIRI samples. Edge width scales with interaction strength. Mononuclear phagocytes show dominant signaling flux with endothelial cells and NK/T cells (e.g., ligand-receptor pairs: MIF-(CD74+CXCR4)). **(E, F)** Receptor-ligand pairing: Cell-type-specific interactions (e.g., endothelial→B cells MIF signaling). **(G)** Cell-type proportions: Stacked barplot showing mononuclear phagocytes and NK/T cells as predominant populations (>65% combined). HIRI induces significant shifts in mononuclear phagocytes (**P < 0.01 vs. control). **(H, I)** Key gene expression: Uniform Manifold Approximation and Projection (UMAP) plots of ATF3, TNFAIP3, IL1B, and KDM6B enrichment in mononuclear phagocytes (log-fold change >2.0 vs. other types). **(J)** Quantitative comparison: Violin plots confirm the highest composite key gene expression in mononuclear phagocytes. ATF3, TNFAIP3, and KDM6B show significant HIRI-induced upregulation (*P* < 0.05, Wilcoxon). *Analysis tools: CellPhoneDB v4.0 (interaction networks), scType (annotation validation). Proportion significance: Chi-square test. Expression thresholds: FDR-adjusted P<0.05. Cell populations defined by: LYZ+ (mononuclear phagocytes), CD3D+/NKG7+ (NK/T cells), VWF+ (endothelial), MZB1+ (plasma cells), CD79A+ (B cells)*.

Intercellular communication analysis demonstrated that mononuclear phagocytes exhibited significantly more interactions with endothelial cells and NK/T cells than other cell populations in both HIRI and control conditions. Moreover, the interaction intensity between mononuclear phagocytes and NK/T cells was relatively high (**Figures 10A-D**). This indicated that, compared with other cell types, mononuclear phagocytes might play a more important role in HIRI. Receptor-ligand pairing patterns across distinct cell populations were comparatively visualized for HIRI and control samples in **Figures 10E, F**. For example, in both HIRI and control samples, the interactions between endothelial cells and B cells, as well as between plasma cells and B cells, were mediated by MIF-(CD74+CXCR4). Cell type composition analysis of the GSE171539 dataset revealed mononuclear phagocytes and NK/T cells as predominant populations across both experimental groups, with notable inter-group proportional variations (**Figure 10G**). Subsequently, the expression levels of ATF3, TNFAIP3, IL1B, and KDM6B at the cell level were investigated. The results showed that ATF3, TNFAIP3, IL1B, and KDM6B all had high expression levels in mononuclear phagocytes (**Figures 10H, I**). Furthermore, among these 5 cell types, the comprehensive expression level of key genes in mononuclear phagocytes ranked the highest, and the expression levels of ATF3, TNFAIP3, and KDM6B in mononuclear phagocytes differed significantly between HIRI and control samples (P < 0.05) (**Figure 10J**). Therefore, mononuclear phagocytes were identified as key cells.

### 3.10 Differentiation states of mononuclear phagocytes and expression alterations of key genes were investigated

**Figure 11:**
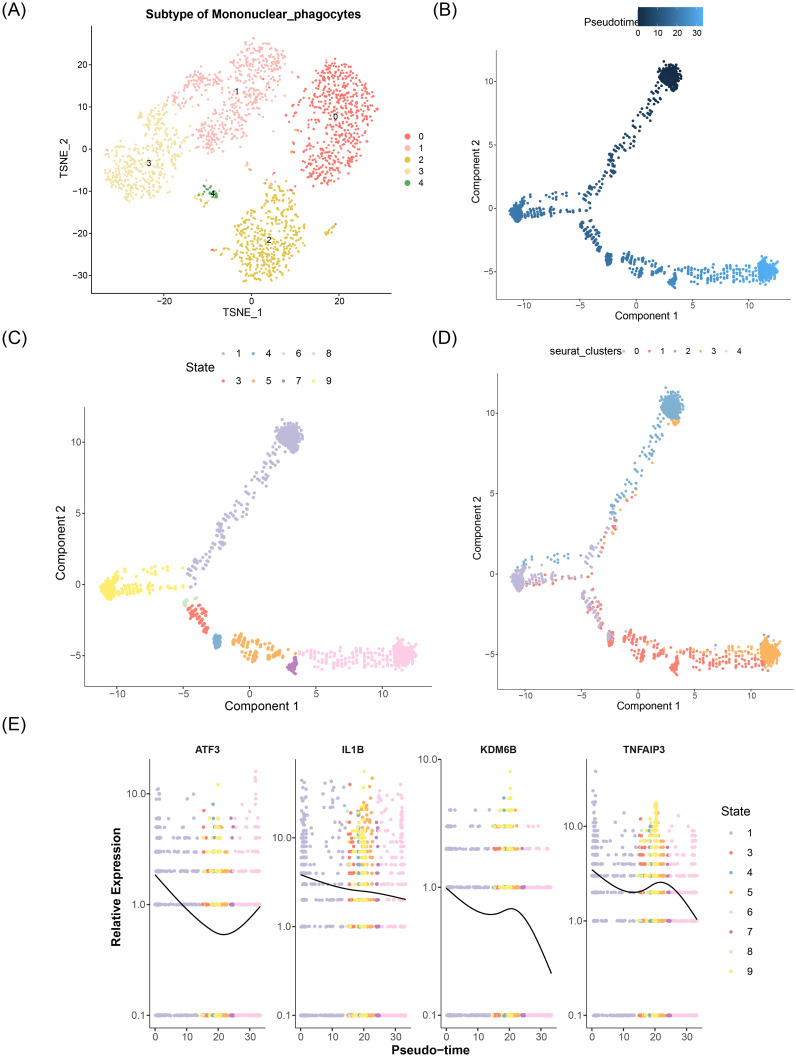
Differentiation trajectories and key gene dynamics in mononuclear phagocytes during HIRI. **(A)** Subcluster identification: Mononuclear phagocytes (annotated per LYZ/CD68 expression) resolved into 5 transcriptionally distinct subclusters (Monocle3 v1.3.4). **(B)** Developmental trajectory: Pseudotemporal ordering (reversed graph embedding) reveals 9 trajectory states (State 1: earliest differentiation). **(C)** State distribution: UMAP colored by trajectory states. **(D)** Pseudotemporal progression: Subcluster distribution along trajectory (blue gradient: early→late differentiation). Subcluster 2 originates earliest but lacks state specificity (expression dispersion >2.0). **(E)** Key gene kinetics: Dynamic expression of ATF3 (biphasic; *P* < 0.01 segmented regression), TNFAIP3 (gradual decline; r=−0.67, P = 3.2e^-5^), IL1B/KDM6B (multiphasic changes; *P* < 0.05 ANOVA) across differentiation. *Trajectory inference: Monocle3 pseudotime analysis with DDRTree reduction. Gene expression modeling: LOESS regression (span=0.75). Statistical thresholds: State* sp*ecificity ≥1.5-fold expression variance across subclusters. Methods validated per hepatic myeloid differentiation frameworks*.

A secondary dimensionality reduction clustering analysis was conducted on key cells, and mononuclear phagocytes were categorized into 5 subtypes (**Figure 11A**). Pseudotemporal ordering analysis positioned mononuclear phagocyte subtypes along a developmental continuum based on differentiation progression. Pseudotime analysis revealed a colorimetric gradient where deeper blue hues indicated earlier differentiation stages. Mononuclear phagocytes exhibited nine distinct differentiation states, with State 1 representing the most primitive and lineage-specific phase (**Figures 11B, C**). Mononuclear phagocytes also included five differentiation subtypes, with subtype 2 differentiating the earliest but not being specific (**Figure 11D**). During the differentiation of mononuclear phagocytes, the expression level of ATF3 decreased initially and then rose; the expression of TNFAIP3 exhibited a general downward trend, while the expression levels of IL1B and KDM6B decreased first, then increased, followed by a sharp decrease (**Figure 11E**). These results indicated that the expressions of ATF3, IL1B, and KDM6B might have dynamic and non-linear change characteristics during the differentiation process of mononuclear phagocytes, which might be related to the biological functions they play at different differentiation stages.

### 3.11 The expression of key genes was verified

**Figure 12:**
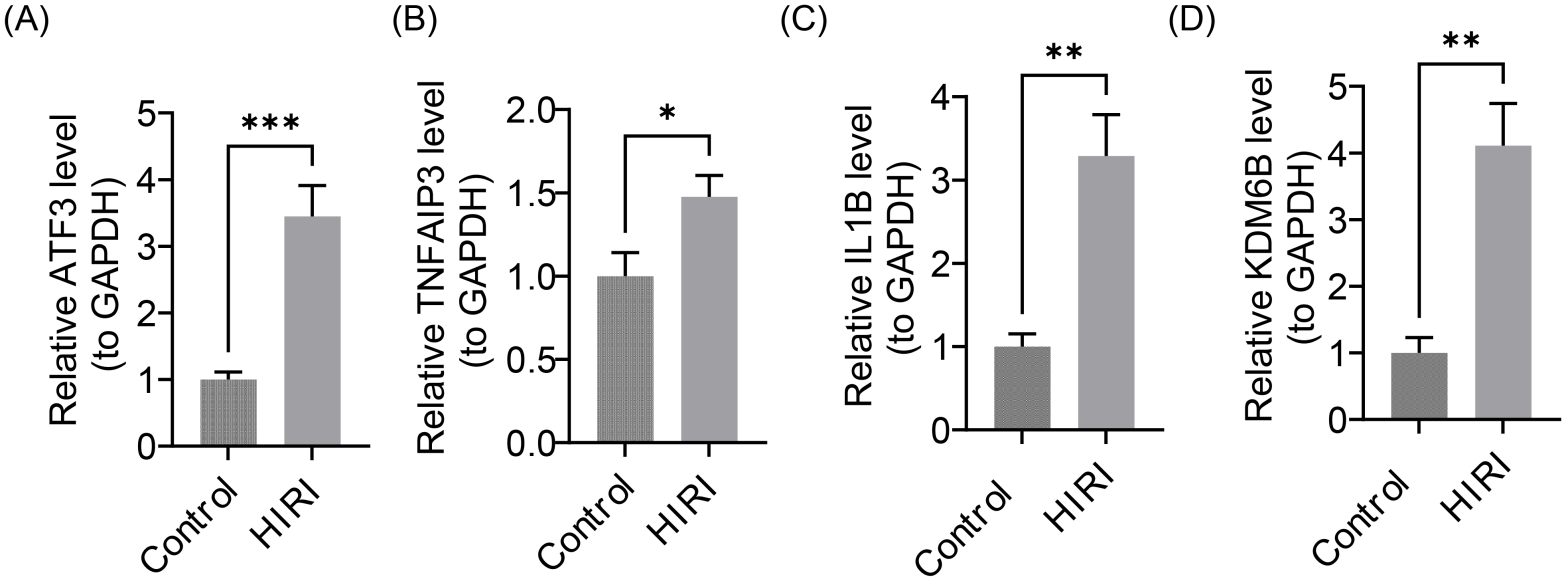
Validation of the expression of key genes in HIRI animal samples. **(A)** ATF3; **(B)** TNFAIP3; **(C)** IL1B; **(D)** KDM6B (*P < 0.05, **P < 0.01, ***P < 0.001). This was consistent with the results of bioinformatics analysis of the GSE12720 and GSE14951 datasets.

Previous bioinformatics analyses have shown that ATF3, TNFAIP3, IL1B, and KDM6B were up-regulated in HIRI samples in the GSE12720 and GSE14951 datasets (P < 0.05). This led to the further use of RT-qPCR methods to verify the expression levels of these key genes in HIRI animal samples. The RT-qPCR results demonstrated that ATF3, TNFAIP3, IL1B, and KDM6B were up-regulated in HIRI animal samples (P < 0.05) (**Figures 12A–D**), which was congruent with the results in the GSE12720 and GSE14951 datasets, verifying the accuracy of the bioinformatics analysis.

## 4 Discussion

HIRI is a common clinical disorder in contexts like hepatectomy and liver transplantation, often resulting in acute liver failure and markedly affecting the prognosis of individuals with liver conditions (33–35). A core pathological characteristic of HIRI is regulated cell death, among which metabolic cell death —a subset of regulated cell death comprising ferroptosis, cuproptosis, disulfidptosis, lysozincrosis, and alkaliptosis (6) —plays a pivotal role. Importantly, ferroptosis and cuproptosis are closely linked to HIRI: ferroptosis is triggered by lipid peroxidation, while cuproptosis—a new type of cell death reliant on mitochondrial respiration—has been shown to participate in ischemia-reperfusion injury in critical organs like the heart and brain (36–38), thus suggesting their potential as therapeutic targets. This study identified four key genes (ATF3, TNFAIP3, IL1B, and KDM6B) associated with MCD in the context. Mononuclear phagocytes were identified as the key cell type through which these genes exert their function for HIRI.

The nomogram model constructed based on key genes demonstrated excellent predictive performance, particularly suitable for risk stratification and perioperative management of high-risk patients in hepatic surgery. Based on the total model score (ranging from 0 to 100 points), patients can be classified into three categories: low-risk (<40 points), intermediate-risk (40–60 points), and high-risk (>60 points). Among high-risk patients (total score >60 points), the probability of hepatic ischemia-reperfusion injury (HIRI) exceeds 80%, and an intensified intervention strategy is recommended, including ischemic preconditioning and postoperative antioxidant therapy (e.g., N-acetylcysteine) (39, 40). Low-risk patients may follow standard surgical procedures to avoid overtreatment.

If the nomogram indicates high-risk status and intraoperative ultrasound reveals a resistance index (RI) >0.7 in the left hepatic lobe (suggesting aggravated ischemic stress), it is advised to shorten the ischemia time from 30 minutes to 20 minutes and adopt an intermittent ischemia strategy to alleviate key gene-mediated metabolic cell death (such as the inflammatory cascade triggered by IL1B) (41, 42). During surgery, controlled slow reperfusion can be achieved via a micro-pump system at a rate of 5 mL/min, with real-time monitoring of key gene expression levels. Should IL1B expression increase more than 2-fold compared to the preoperative level, additional anti-inflammatory treatment, such as an IL-1 receptor antagonist, should be administered (43).

Furthermore, for high-risk patients, key gene expression and liver function indicators should be assessed at 6, 12, and 24 hours postoperatively. If the nomogram-predicted probability of HIRI increases by more than 15% compared to the preoperative level, or if ATF3/TNFAIP3 expression increases 1.5-fold relative to the 6-hour postoperative level, early intervention should be initiated even if alanine aminotransferase (ALT) levels are not significantly elevated (<200 U/L).

This nomogram model can also be integrated into existing clinical workflows as a key component of a “postoperative liver function early-warning system.” For example, if a patient meets both criteria of “IL1B expression increased more than 3-fold” and “ALT >300 U/L,” the system can automatically alert the attending physician, prompting the initiation of combined “anti-inflammatory and antioxidant” therapy to effectively reduce intervention delays.

In summary, the nomogram model developed in this study has the potential to serve as a standardized tool for HIRI risk assessment in hepatic surgery, facilitating precise perioperative management and reducing the incidence and severity of HIRI.

ATF3, a basic leucine zipper transcription factor, represents a stress-responsive regulator potentially implicated in HIRI pathology (35). This study demonstrated significantly elevated ATF3 expression in HIRI models through RT-qPCR validation, consistent with its dynamic response to reperfusion stress. The observed correlation between ATF3 and TNFAIP3 suggests potential cooperative interactions in modulating inflammatory responses. Functional enrichment analyses further associated ATF3 with pathways relevant to cellular adaptation under oxidative stress conditions. Within the context of regulated cell death mechanisms, MCD, including ferroptosis and cuproptosis, constitutes an important pathological component in HIRI (7). Studies have found that activation of ATF3 may regulate the expression or activity of GPX4, ultimately affecting the occurrence of ferroptosis and inhibiting the progression of hepatocellular carcinoma (44). Furthermore, upregulation of ATF3 can downregulate the transcription of GPX4, promote ferroptosis, and inhibit the proliferation of gastric cancer cells, providing a potential therapeutic target for gastric cancer (45). During myocardial ischemia/reperfusion injury (MI/RI), ATF3 promotes ferroptosis by upregulating the expression of ACSL4, leading to aggravated cardiomyocyte damage. Therefore, inhibiting the expression of ATF3 or ACSL4 may help alleviate ferroptosis and mitigate the effects of MI/RI (46). Additionally, ATF3 can activate the transcription of RNF146, promoting the ubiquitination and degradation of ACSL4 by RNF146, thereby indirectly downregulating ACSL4 and inhibiting ferroptosis (47). Simultaneously, ATF3 enhances cellular antioxidant capacity by regulating the expression of SLC7A11, alleviating intestinal ischemia-reperfusion injury and inhibiting ferroptosis (48). In endometriosis, IL-33/ST2 inhibits ATF3 by activating the p38/JNK signaling pathway, thereby upregulating the expression of SLC7A11, reducing ferroptosis, and protecting endometriotic stromal cells (eESCs) from damage (49). Transcription factors like ATF3 may contribute to cellular defenses against such metabolic death pathways, though the precise molecular interactions require experimental validation. Future studies should establish whether ATF3 modulates specific MCD subroutines (e.g., ferroptosis through lipid peroxidation pathways or cuproptosis via mitochondrial respiration regulation) and clarify its functional relationships with other key regulators like TNFAIP3. Studies have shown that toxic products generated from lipid peroxidation can directly damage mitochondria, leading to deterioration of mitochondrial function, and mitochondrial damage in turn exacerbates lipid peroxidation, forming a vicious cycle that further aggravates cellular damage (50). This mechanism indicates that lipid peroxidation and mitochondrial dysfunction interact with each other, promoting oxidative stress and cell death (51). However, whether ATF3 acts as an upstream driver or merely a parallel event in this process still requires further exploration.

TNFAIP3 (A20), a critical ubiquitin-editing enzyme, regulates immune modulation, apoptosis, and inflammatory responses by negatively regulating NF-κB signaling pathways. Mutations in this gene are linked to various immune disorders (e.g., autoimmune hepatitis, rheumatoid arthritis), reflecting its key role in balancing immune homeostasis (52, 53). In HIRI, TNFAIP3 functions as a double-edged regulator: under physiological conditions, its inhibition of NF-κB restricts excessive inflammatory responses, thereby reducing hepatocellular damage; however, when its expression or ubiquitination is dysregulated (52, 54), this inhibitory effect is disrupted—either insufficiently suppressing NF-κB (leading to hyperinflammation) or overly dampening protective anti-inflammatory signals. Notably, genetic knockout of TNFAIP3 eliminates NF-κB restraint entirely, triggering massive release of pro-inflammatory cytokines (e.g., TNF-α, IL-1β) and enhancing inflammatory cell infiltration into the liver, which exacerbates HIRI. This context-dependent duality—mediated by ubiquitination-dependent regulation of NF-κB—highlights TNFAIP3 as a pivotal therapeutic target, emphasizing the need to precisely modulate its activity to balance pro- and anti-inflammatory responses in HIRI. Studies have shown that toxic products generated from lipid peroxidation can directly damage mitochondria, leading to deterioration of mitochondrial function, and mitochondrial damage in turn exacerbates lipid peroxidation, forming a vicious cycle that further aggravates cellular damage (50). This mechanism indicates that lipid peroxidation and mitochondrial dysfunction interact with each other, promoting oxidative stress and cell death (51). However, whether ATF3 acts as an upstream driver or merely a parallel event in this process still requires further exploration.

Our study confirmed its significant upregulation in HIRI samples via RT-qPCR, consistent with transcriptomic data in GSE12720 and GSE14951, and single-cell analysis further revealed high expression in mononuclear phagocytes—validating these cells as a key source of IL1B in HIRI. Interleukin-1 beta (IL1B) is a potent proinflammatory cytokine that amplifies hepatic inflammatory cascades in HIRI by inducing chemokines (e.g., CXCL5) and secondary cytokines (e.g., IL-6, TNF-α) (55). In HIRI pathogenesis, IL1B maturation is triggered via neutrophil-derived proteases (e.g., elastase) and macrophage-expressed NLRP3 inflammasomes, creating a feedforward loop that drives hepatocellular damage (56, 57). Furthermore, studies have shown that IL-1β activates the NLRP3 inflammasome through its pro-inflammatory effects, further exacerbating the inflammatory response (58). The NLRP3 inflammasome also regulates ferroptosis in asthma inflammation through the JAK2/STAT3 pathway (59). Therefore, IL-1β may also play an important role in metabolic cell death, and this hypothesis requires further experimental validation. Single-cell analysis additionally showed that IL1B is highly expressed in mononuclear phagocytes—recognized as key cells in HIRI—which enhances its proinflammatory effects via crosstalk with liver sinusoidal endothelial cells and NK/T cells (60). This cell-crosstalk mechanism (neutrophil-macrophage axis) enables spatial amplification of IL1B signaling, resulting in sustained inflammation and parenchymal injury (57, 61). Notably, our correlation analysis showed IL1B is positively associated with ATF3 and TNFAIP3, suggesting a coordinated regulatory network in modulating pyroptosis and inflammatory responses in HIRI. Therapeutic strategies targeting IL1B maturation (e.g., NLRP3 inhibitors) or its downstream effectors show promise in mitigating HIRI progression, positioning IL1B as both a biomarker and actionable target in clinical management.

KDM6B functions as a critical histone demethylase catalyzing the demethylation of H3K27me2/3, thereby epigenetically regulating gene expression (6). Its dysregulation has been involved in impairing the activation and polarization of immune cells, especially in macrophages, and may promote pro-inflammatory responses that play a key role in the pathogenesis of ischemia-reperfusion injury (6, 33–35). This study identified KDM6B as a key regulatory gene in HIRI. Single-cell sequencing analysis demonstrated a biphasic expression pattern of KDM6B in the differentiation process of mononuclear phagocytes (MNPs): increased expression at early stages may drive the transcription of pro-inflammatory genes, whereas in later stages, its expression shifts to participating in the activation of repair-related genes.

Functional enrichment analyses further suggested a potential link between KDM6B activity and oxidative phosphorylation pathways, consistent with its plausible role in modulating stress responses relevant to cell death mechanisms such as ferroptosis (6). Moreover, in this study, KDM6B expression also showed a positive correlation with ATF3 and TNFAIP3, with co-enrichment observed in pathways such as olfactory transduction, hinting at potential cooperative roles in hepatic metabolic stress adaptation. Molecular docking analysis identified bisphenol A as a potential high-affinity ligand for KDM6B (ΔG < -5 kcal/mol), suggesting its potential to modulate KDM6B’s demethylase activity and macrophage function, thus offering a novel avenue for therapeutic exploration in HIRI. Current research gaps include the need for direct functional validation of KDM6B in HIRI models, elucidation of its specific target genes in MCD pathways, and confirmation of cell-type-specific regulatory mechanisms. Future research should focus on comprehensively delineating KDM6B ‘s epigenetic regulatory network in HIRI and evaluating its translational potential as a therapeutic target.

GSEA revealed that core genes ATF3, TNFAIP3, IL1B, and KDM6B significantly converged on NOD-like receptor signaling, spliceosome, and olfactory transduction pathways (**Figures 5A-D**), providing insights into how these genes coordinately regulate MCD and inflammatory responses in HIRI. Complementary evidence from the GGI network highlighting TAX1BP1’s role in interleukin-1 response regulation (**Figure 5E**). These findings are contextualized by existing literature on HIRI pathophysiology. NOD-like receptor signaling: Prior studies established that this pathway triggers NLRP3 inflammasome activation and IL-1β maturation in Kupffer cells, directly contributing to hepatocellular damage during reperfusion (62). Our results further confirm this mechanism by demonstrating ATF3/TNFAIP3/IL1B co-enrichment (False Discovery Rate (FDR) <0.001), reinforcing its central role in sterile inflammation. 2. Spliceosome regulation. Spliceosome regulation: Hypoxia-induced alternative splicing has been implicated in cellular stress adaptation, particularly through remodeling of HIF-1α isoforms that modulate oxygen-sensing pathways (63). Our identification of ATF3/TNFAIP3/IL1B enrichment (NES >1.8; p<0.001) in spliceosome pathways reveals its potential significance in post-transcriptional regulation during hepatic reperfusion stress. Olfactory transduction: While traditionally associated with sensory neurons, emerging evidence indicates olfactory receptors (e.g., OR1A1) participate in hepatic metabolic reprogramming and inflammatory responses (64). The co-enrichment of ATF3/TNFAIP3/KDM6B in this pathway suggests a novel axis for hepatocyte stress sensing in HIRI. Studies have demonstrated that OR1A1, which is expressed in hepatocytes, does not directly participate in olfactory perception but functions as a metabolic sensor detecting endogenous metabolites or exogenous stress signals (65, 66). Key signaling molecules involved in the olfactory transduction pathway, such as olfactory receptors, cyclic adenosine monophosphate (cAMP), and phosphodiesterases, are also found in the liver (67, 68), and through the modulation of the cAMP-PKA signaling axis, these molecules are involved in hepatic antioxidative stress response and maintenance of mitochondrial function, particularly playing a significant role in HIRI (69, 70). Furthermore, the activation of OR1A1 may enhance the expression of genes related to fatty acid β-oxidation, thereby alleviating lipid accumulation caused by mitochondrial dysfunction during HIRI (66). Dysregulated lipid metabolism constitutes a core trigger of ferroptosis (71). The common enrichment of ATF3, TNFAIP3, and IL1B within the olfactory transduction pathway suggests their potential as core drivers of “metabolic cell death,” although further experimental validation is required. These pathways functionally intersect at inflammation-immune regulation, as evidenced by TAX1BP1 - a regulator of NF-κB signaling (72) -which interacts with TNFAIP3 and IL1B to connect interleukin-1 responses to transcriptional control of MCD-related genes. The NOD-like receptor pathway remains the best-characterized mechanism in HIRI (62), while the spliceosome and olfactory pathways represent emerging therapeutic targets warranting experimental validation.

Immune cell profiling demonstrated a robust association between activated CD4+ T cell abundance and ATF3 expression (Pearson’s r=0.88, P<0.05), consistent with their established involvement in HIRI development. Activated CD4+ T cells drive liver damage through IFN-γ-dependent macrophage polarization that amplifies hepatocellular necroptosis (61), IL-17-mediated neutrophil recruitment via hepatocyte-derived CXCL5 (73), and direct Fas/FasL cytotoxic signaling (inducing apoptosis) (74). The strength of this correlation suggests ATF3, highly expressed in mononuclear phagocyte, may promote secretion of CXCL5 by these cells to upregulate chemokine receptors (e.g., CXCR3) on CD4^+^ T cells, facilitating their infiltration Conversely, KDM6B exhibited a significant negative correlation with CD56bright NK cells (r = -0.46, P < 0.05), and this could indicate regulatory cytolytic roles that restrict early-phase injury (75). Mast cell-activated CD4+ T cell interactions (r = 0.85, P < 0.05) further amplify inflammation via IL-4/IL-33 crosstalk (73), while therapeutic CD4+ T cell depletion reduces transaminases by >65% (75), reinforcing their translational relevance.

Molecular docking revealed strong binding affinities (ΔG < -5 kcal/mol) between core HIRI-related genes and predicted compounds: ATF3, TNFAIP3, IL1Band KDM6B, with trovafloxacin exhibiting exceptional binding to TNFAIP3 (ΔG = -10.7 kcal/mol). These drugs demonstrate disease-relevant mechanisms: 1. Resveratrol (targeting IL1B). Established role: Reduces liver necrosis by 41% in IRI models through SIRT1-mediated NLRP3 inflammasome suppression (21). Clinical relevance: Validated hepatoprotectant against IL1β-driven inflammation. 2. Trovafloxacin (targeting TNFAIP3).Paradoxical action: Antibiotic with documented hepatotoxicity via mitochondrial apoptosis (76).Novel insight: High-affinity TNFAIP3 binding suggests potential NF-κB modulation 3.Deoxynivalenol (targeting ATF3): Mechanistic link: Triggers ATF3-mediated Endoplasmic Reticulum (ER) stress in enterocytes (77). First-reported potential: May activate adaptive stress responses in hepatocytes 4. Bisphenol A (targeting KDM6B): Epigenetic regulation: Alters KDM6B-dependent H3K27 demethylation in macrophages (78). Innovative implication: Dual targeting (ATF3/KDM6B) suggests immunomodulatory synergy. Notably, bisphenol A, as an environmental toxin, has been extensively studied and is known to have potential hazards to humans and animals, such as endocrine disruption and organ toxicity (79, 80). Therefore, when considering it as a therapeutic candidate, its potential risks must be fully considered. The current molecular docking results are only a preliminary screening basis, and the biological effects of the drug still require further exploration. While resveratrol remains clinically promising for HIRI (81), deoxynivalenol and bisphenol A represent novel candidates requiring experimental validation of their hepatic protective effects.

Notably, this study identified regulators of metabolic cell death (IL1B, KDM6B), providing potential translational directions for the transplantation field. Resveratrol (targeting IL1B) mildly suppresses T-cell overactivation through SIRT1 pathway activation, thereby preventing rejection, and demonstrates no nephrotoxicity compared to traditional immunosuppressants (82). Thus, resveratrol may serve as an “adjunct immunosuppressive drug” combined with low-dose calcineurin inhibitors to both reduce rejection risk and alleviate hepatic ischemia-reperfusion injury (HIRI) (83). Additionally, bisphenol A targets KDM6B, a histone demethylase that balances immune responses by modulating the epigenetic state of macrophages (84). Future development of KDM6B inhibitors may reduce the proportion of pro-inflammatory M1 macrophages while enhancing anti-inflammatory M2 macrophage function, thereby diminishing immune rejection and effectively mitigating infection risks.

The traditional University of Wisconsin (UW) preservation solution fails to effectively suppress metabolic cell death induced by cold ischemia-reperfusion during organ preservation (85). This study proposes adding resveratrol and trovafloxacin (a TNFAIP3 activator) to the preservation solution. Research demonstrates that resveratrol significantly prolongs survival after liver transplantation in Wistar rats and alleviates ischemia-induced fat deposition, necrosis, and apoptosis (86). Furthermore, TNFAIP3 as an NF-κB inhibitor may help suppress the overexpression of cuproptosis-related genes and reduce cold ischemia-induced mitochondrial damage (87, 88). Regarding translational ethics, three major challenges exist: First, trovafloxacin may cause hepatotoxicity related to mitochondrial apoptosis, necessitating pre-experimental dose escalation (0.1-2.0 mg/kg) to establish a safe dosage; Second, prioritizing FDA-approved resveratrol for clinical trials is recommended to minimize risks; Finally, informed consent documents in clinical trials should clearly disclose potential metabolic side effects (e.g., blood glucose fluctuations) and annual liver/kidney function follow-up requirements to ensure participant understanding and consent.

The scRNA-seq enables unprecedented resolution of liver cellular heterogeneity during HIRI, revealing specialized functional subsets within MNPs that coordinate injury progression and repair (89). Our identification of MNPs aligns with their dual roles in HIRI: 1) Early pro-inflammatory activation, where recruited monocytes differentiate into TNF-α/IL-1β-producing macrophages that amplify neutrophil infiltration and parenchymal damage (90), and 2) Late reparative reprogramming, where TREM2+ macrophages emerge to resolve inflammation through efferocytosis and pro-regenerative cytokine secretion. Pseudotemporal trajectory analysis of MNP subtypes indicates progressive differentiation from classical monocytes (CD14+ CCR2+). These cells differentiate toward either inflammatory (CD86hi MHC-IIhi) or anti-inflammatory (CD163+ CD206+) terminal states, with key genes exhibiting stage-specific expression dynamics: ATF3 peaks in transitional MNPs during the oxidative stress phase (< 6 h post-reperfusion), regulating ER stress adaptation (89), while TNFAIP3 progressively increases in reparative MNPs (> 24 h), suppressing NF-κB to facilitate inflammation resolution (90). This temporal regulation suggests that ATF3 governs initial damage responses, whereas TNFAIP3 promotes recovery - a differentiation-dependent mechanism where MNPs evolve from damage sensors to regeneration coordinators (89, 89).

The single-cell analysis in this study not only identified MNPs as a key cell population in HIRI but also revealed the dynamic, non-linear expression patterns of ATF3, IL1B, and KDM6B during cell differentiation through pseudotime trajectory analysis. These changes may be closely related to the functional specialization of MNPs at different differentiation stages: early high expression of ATF3 and IL1B may promote polarization toward a pro-inflammatory (M1-like) phenotype (91, 91), enhancing the initial response to ischemia-reperfusion injury; whereas, as differentiation progresses, fluctuations in KDM6B expression may participate in epigenetic reprogramming, guiding the transition to a reparative (M2-like) phenotype (92), promoting inflammation resolution and tissue repair. These findings suggest that key genes may play a dual role in the initiation and resolution phases of HIRI inflammation by regulating the functional state transition of MNPs. Furthermore, the fluctuations in key gene expression observed in pseudotime analysis highly align with the differentiation process of MNPs from classical monocytes to inflammatory or reparative macrophages (93). The increase in ATF3 during early differentiation stages may be consistent with its role as a stress-responsive transcription factor, participating in adaptive responses to endoplasmic reticulum stress and oxidative stress (94); while the peak expression of IL1B coincides with the activation phase of the NLRP3 inflammasome, indicating its key role in driving the transition to a pro-inflammatory state (95). Changes in KDM6B expression may influence gene expression related to metabolic reprogramming and inflammation resolution by regulating H3K27me3 modification, thereby promoting the shift to a reparative phenotype in later stages. These findings link the expression dynamics of key genes to transitions in cell functional states, providing new perspectives for understanding the spatiotemporal regulation of immune cells in HIRI.

Additionally, this study uncovered the central role of MNPs in intercellular communication in the liver through cell communication analysis, particularly under HIRI conditions, where the cell communication network undergoes significant reprogramming. In HIRI samples, interactions between MNPs and other cells, such as endothelial cells and NK/T cells, were significantly enhanced, indicating that the injury environment promotes ‘crosstalk’ between cells. In terms of ligand-receptor interactions, although the MIF-(CD74+CXCR4) pathway was active in both control and HIRI groups, under HIRI conditions, this signaling was primarily concentrated between MNPs and NK/T cells, potentially promoting lymphocyte recruitment and activation, consistent with the increased CD4+ T cells observed in immune infiltration analysis. More critically, these communication changes are closely related to key genes. For example, the upregulation of the pro-inflammatory factor IL1B highly expressed in MNPs under HIRI may amplify inflammatory responses toward hepatic sinusoidal endothelial cells and T cells through the IL1B-IL1R1 signaling axis (96, 97), providing a cellular mechanism explanation for the role of IL1B in local inflammation. Simultaneously, changes in the expression of TNFAIP3, a negative regulator of NF-κB signaling, in MNPs may indirectly affect cell death pathways dependent on TNF superfamily ligand-receptor interactions by modulating the intensity of inflammatory signal responses (98, 98). Therefore, HIRI not only alters the expression of key genes but also reshapes the cell communication microenvironment centered on MNPs through these genes, creating an environment inclined toward pro-inflammation and cell death, which may be a key mechanism driving HIRI progression.

This study identified ATF3, TNFAIP3, IL1B, and KDM6B as core regulators of metabolically dysregulated cell death in HIRI, validated through a high-performance nomogram model (AUC >0.90) for clinical risk stratification (99, 99). Functional enrichment revealed these genes converge on NOD-like receptor signaling (promoting NLRP3 inflammasome-mediated pyroptosis) ^[11]^, spliceosome pathways (remodeling hypoxia-responsive transcripts) (99), and olfactory transduction (modulating non-canonical metabolic sensing). Single-cell resolution demonstrated dynamic expression in pericentral hepatocytes – the zone most sensitive to I/R damage – and monocyte-derived macrophages that orchestrate inflammation-resolution balance via endothelial crosstalk (60). These results provide novel mechanistic insights into spatial-temporal injury progression (100), potentially informing targeted therapies like engineered exosomes modulating the gp78-ACSL4 ferroptosis axis or BMSC-derived miRNA delivery (101).

The primary limitation of this study lies in the insufficient translational validation, as relevant verification has not yet been conducted in clinical samples, and significant heterogeneity exists in the IRI transcriptome among patients (69, 70). Future research should focus on validating gene functions in clinical samples, developing cell-type-specific delivery systems, and analyzing spatial metabolic vulnerabilities (68). Additionally, this study did not establish a causal relationship between key genes and metabolic cell death, and drug predictions lack experimental validation (71). Therefore, future work should involve *in vitro* and *in vivo* experiments to further confirm the roles of these genes in cell death and verify the biological effects of predicted drugs (65, 66). Moreover, the role of mononuclear phagocytes in metabolic cell death remains unverified, and the low resolution of single-cell RNA-seq limits the precise delineation of cell populations (68). Future studies will integrate spatial transcriptomics to obtain more detailed regional heterogeneity information and employ experiments such as flow cytometry and lineage-specific markers to further validate the functions of mononuclear phagocytes. Furthermore, dataset heterogeneity and batch effects may compromise model accuracy and generalizability; these issues could be addressed through batch effect correction, ensemble learning methods, and increased sample sizes. Finally, the interpretability of machine learning models is limited; future efforts could incorporate techniques such as Shapley Additive Explanations (SHAP) and Local Interpretable Model-agnostic Explanations (LIME) to enhance the transparency and trustworthiness of the findings.

## Data Availability

This study systematically explores the role of metabolism-related cell death genes (MRGs) in hepatic ischemia-reperfusion injury (HIRI), a condition with profound immune and metabolic involvement. By integrating three independent transcriptomic datasets (GSE12720, GSE14951, and GSE171539) with a curated list of 478 MRGs, we employed differential expression analysis, multiple machine learning algorithms, and experimental validation to identify ATF3, TNFAIP3, IL1B, and KDM6B as core regulators. Functional enrichment and immune infiltration analyses revealed strong associations between these genes and inflammatory pathways, including activated CD4 T cells and mononuclear phagocytes. In addition, nomogram construction demonstrated their predictive potential for HIRI occurrence, while drug prediction and molecular docking highlighted candidate therapeutic agents with strong binding affinities. Single-cell RNA sequencing further uncovered dynamic gene expression changes during immune cell differentiation, and RT-qPCR confirmed their up-regulation in animal models. Collectively, this work uncovers the molecular and immunological mechanisms of metabolic cell death in HIRI, identifies biomarkers and therapeutic targets, which falls within the scope of *”Frontiers in Immunology”* in the field of immune-related disease mechanisms.
