# Supplementary material for: Identification of key genes related to metabolic cell death in hepatic ischemia-reperfusion injury from transcriptome data and mechanism research using single-cell data
Source: Front Immunol. 2026 Jan 2;16:1695979. doi: 10.3389/fimmu.2025.1695979 (PMC12808398; doi:10.3389/fimmu.2025.1695979)
Supplement: Supplementary file 1 [file Table1.docx]

Supplementary Material

# Supplementary Tables

**Supplementary Table 1.** MRGs

**Supplementary Table 2.** Cell types and their marker genes

**Supplementary Table 3.** Primer sequences for key genes and GAPDH

**Supplementary Table 4.** GO terms

**Supplementary Table 5.** KEGG pathways

**Supplementary Table 6.** GSEA results

**Supplementary Table 7.** miRNAs of key genes

**Supplementary Table 8.** Drug prediction results
