# Supplementary material for: Identification of key genes related to metabolic cell death in hepatic ischemia-reperfusion injury from transcriptome data and mechanism research using single-cell data
Source: Front Immunol. 2026 Jan 2;16:1695979. doi: 10.3389/fimmu.2025.1695979 (PMC12808398; doi:10.3389/fimmu.2025.1695979)
Supplement: Supplementary file 3 [file Table3.docx]

**Table S2 Cell types and their marker genes**

| Cell types | marker genes |
| --- | --- |
| Mononuclear_phagocytes | CD68 |
| NK/T_cells | CD3D、FCGR3A |
| B_cells | MS4A1 |
| Plasma_cells | SDC1 |
| Endothelial_cells | PECAM1 |
